# Influence of the surgical method used for lip closure on the shape of the maxillary arch: a retrospective 3D analysis of plaster models

**DOI:** 10.1186/s13005-025-00550-5

**Published:** 2025-10-28

**Authors:** Michaela Buckova, Christiane Keil, Stefan Holtzhausen, Phillip Sembdner, Tom Alexander Schröder, Theodosia Bartzela, Franz Tritschel, Milan Drahos, Günter Lauer

**Affiliations:** 1 Department of Oral Maxillofacial Surgery, University Hospital Carl Gustav Carus, Technische Universität Dresden, Fetscherstr. 74, Haus 30, Dresden, D-01307 Germany; 2 Department of Orthodontics, University Hospital Carl Gustav Carus, Technische Universität Dresden, Dresden, Germany; 3 Virtual Product Development, Working Group Reverse Engineering, Technische Universität Dresden, Dresden, Germany; 4 Institute of Biophysics and Informatics, Department of Medical Statistics, First Medical Faculty of Charles University, Prague, Czech Republic

**Keywords:** Cleft lip and palate, Dental models, Maxillary arch development, Customized semi-automatic 3D analysis

## Abstract

**Background:**

Surgical correction of unilateral cleft lip and palate (UCLP) aims to restore normal facial development and function. Achieving symmetry is essential for dental arch development and facial symmetry. The surgical treatment can result in midfacial retrusion, maxillary constriction, and alveolar segment collapse. The aim of this study was to develop a novel 3D analysis method for assessing possible effects on maxillary arch development following different surgical techniques for lip repair in UCLP patients.

**Methods:**

The dimensions of the maxillary arch were analyzed and compared in children with UCLP who had undergone Delaire or Tennison-Randall/Pfeifer surgery before lip and palate closure using digitized dental plaster casts. Two different evaluation methods were used in the study: the 2D analysis was based on linear measurements using reference points in the OnyxCeph3™ software. The semi-automatic 3D analysis was based on the curve-fitting method and our self-developed customized software application (SMP).

**Results:**

No significant differences were observed using the 2D method. However, the 3D analysis detected statistically significant differences in the Q coefficient (*p* = 0.001393) between the two surgical techniques, demonstrating the superior ability of the 3D method to assess morphological changes.

**Conclusions:**

The 3D analysis did not depend on classical reference points. It provided new information for assessing the collapse of maxillary segments. This analysis is a valuable tool for evaluating the impact of surgical techniques on maxillary arch development in children with UCLP. These findings advocate the integration of advanced 3D imaging tools in cleft surgery planning and postoperative assessment.

**Supplementary Information:**

The online version contains supplementary material available at 10.1186/s13005-025-00550-5.

## Background

Surgical repair of complete unilateral cleft lip and palate (UCLP) may lead to mid-facial hypoplasia due to an incomplete unification of perilabial/perinasal musculature, contributing to malposition and underdevelopment of the underlying bony structures on the affected side [1–4]. In contrast, most non-operated cleft lip patients demonstrate a normal facial projection [5, 6] or an even more protruded maxillary growth compared to normal controls [7]. However, a malposition of the dental arch and a collapse of the segment in the cleft area can also be seen. Therefore, midface hypoplasia may be related to an interaction between disrupted anatomy and normal intrinsic growth mechanisms rather than an initial lack of mesenchymal tissue or abnormal midfacial growth mechanisms [8].

Physiological processes in facial development suggest that UCLPs should be considered displacement malformations rather than defect malformations. Understanding and incorporating these processes into surgical approaches has significantly improved midfacial and nasal growth in cleft lip and palate malformations, leading to optimal outcomes [9]. For many surgical techniques, procedures have been described to improve function, aesthetics and craniofacial development [10–13]. However, surgical closure of cleft lip has been reported to produce unsatisfactory results, especially in unilateral cases, due to maxillary retrusion and downward rotation [3, 14, 15]. Functional closure in accordance with the Delaire technique reduces the maxillary transverse dimensions while maintaining initial sagittal growth [16]. Studies have also shown that the type of lip closure surgery may influence the need for subsequent orthodontic treatment as well as its timing and complexity. Freitas et al. reported that patients who underwent the Millard procedure had a higher incidence of dental abnormalities, such as missing or impacted teeth, compared to those who underwent the Tennison procedure [17]. In addition, the study by Jensen et al. found that patients who underwent the Tennison procedure had a shorter duration of orthodontic treatment than those treated with the Millard procedure [11]. The authors suggest that these differences may be due to the way the surgical approach affects the growth and development of the maxilla and mandible, as well as the position and alignment of the dental arches.

To analyze morphological changes and symmetry in the edentulous maxilla of UCLP patients, two-dimensional (2D) linear evaluation based on reference points on the soft tissues of the jaw segments is commonly used [18, 19]. Using these 2D measurements, it has been shown, among other things, that surgical lip closure reduces the width of the alveolar cleft [20] and that different surgical methods for closing the cleft lip and palate affect the growth of the maxilla to different extents [10]. Analyzes of the symmetry of the affected and unaffected side of the jaw are not yet known because the jaw midline (raphe median plane) is either not available or difficult to determine. 2D model analyzes are inadequate for assessing three-dimensional (3D) morphological changes during maxillary development. In order to analyze the complex 3D structure of the upper jaw, it is therefore necessary to record and analyze the entire surface. This can be done by optical scanning of the surface of jaw impressions [21].

In recent years, digitalization has been introduced in the different treatment modalities of clefts. For example, it is used in fabricating presurgical orthodontic palatal plates in newborns and infants [22–24], in identifying success factors in secondary cleft surgery [25].

The aim of this study is to develop a digital 3D method for describing morphological changes in the jaw of UCLP infants between lip and palate reconstruction and to compare the data with those obtained from a 2D evaluation. In addition, special attention was paid to segmental collapse and the development of the maxillary dental arch under the influence of Delaire and Tennison-Randall/Pfeifer. Therefore, the objectives of this study are as follows:

- 2D analysis of digitized plaster casts based on classic reference points and linear measurements.
- 3D analysis of digitized plaster casts using self-developed custom software application (SMP) based on classical reference points and curve fitting and comparison with 2D analysis.
- Semi-automated 3D analysis of digitized plaster casts using self-developed custom software application (SMP).
- Validation of the method using digitized plaster casts from a defined cohort of patients with UCLP to ensure the accuracy and reliability of the new assessment method in a specific patient population.

## Methods

### Study design

We conducted a retrospective study of children with complete UCLP who underwent surgery at the Department of Oral and Maxillofacial Surgery, University Hospital Carl Gustav Carus, Dresden, Germany, between 2003 and 2022. Both surgical methods for lip closure were performed by a single experienced surgeon. The Delaire procedure was performed between 2009 and 2022 by Prof. Dr. Günter Lauer, and the Tennison-Randall/Pfeifer procedure was performed between 2003 and 2009 by Prof. Dr. Uwe Eckelt. Patients were divided into groups according to the type of surgery. All patients were Caucasian and born at term.

Plaster casts of the patients’ jaws were taken before cleft lip repair (T1) and before palatoplasty (T2). Lip surgery was performed between 4 and 6 months of age, and palate surgery was performed between 9 and 12 months of age. Informed consent was obtained from all parents, and all data were anonymized before analysis. The tenets of the Declaration of Helsinki for medical research involving human subjects were adhered to. The study was approved by the Ethics Committee of the Technical University of Dresden (SR + BO-EK-43012020).

### Patients selection

The dental cast of 59 patients with UCLP were retrospectively evaluated (Table 1). Of these patients, 23 were treated according to Delaire (group D) and 28 according to Tennison-Randall/Pfeifer procedure (group TR/P). The mean age in group 1 was 18.61 ± 4.72 weeks and in group 2 23.1 ± 4.41 weeks (Table 1). Of these 59 patients 118 plaster casts were obtained prior to lip and palate surgery. Six of these casts were excluded due to poor quality impressions and 2 plaster casts were missing. Finally, a total of 102 plaster casts from 51 patients were analyzed in this study.

**Table 1:** Baseline characteristics of the patients

Group	Number of patients	Gender distribution male/female	Mean age (weeks) T1	Mean age (weeks) T2
D	23	12/11	18.61 ± 4.72	43.68 ± 10.50
TR/P	28	18/10	23.1 ± 4.41	50.12 ± 17.73

*D* Delaire procedure, *TR/P* Tennison-Randall/Pfeifer procedure

### Intervention

All plaster models were digitized using a DVT scanner (Morita model MCT-1-EX-1f, Kyoto, Japan) or the *Trios*^®^* 4 Wireless* intraoral scanner (3Shape, Copenhagen, Denmark). These scanners converted the volumetric data into surface geometry models through greyscale segmentation. The geometry is represented as surface-describing triangles, allowing segmentation and marking of different elements of the geometry. This process defines 3D points and 3D polygons, enabling distances measurements. All 3D surface models were exported as Standard Tessellation Language (STL) files.

### Data analysis

**Fig. 1:**
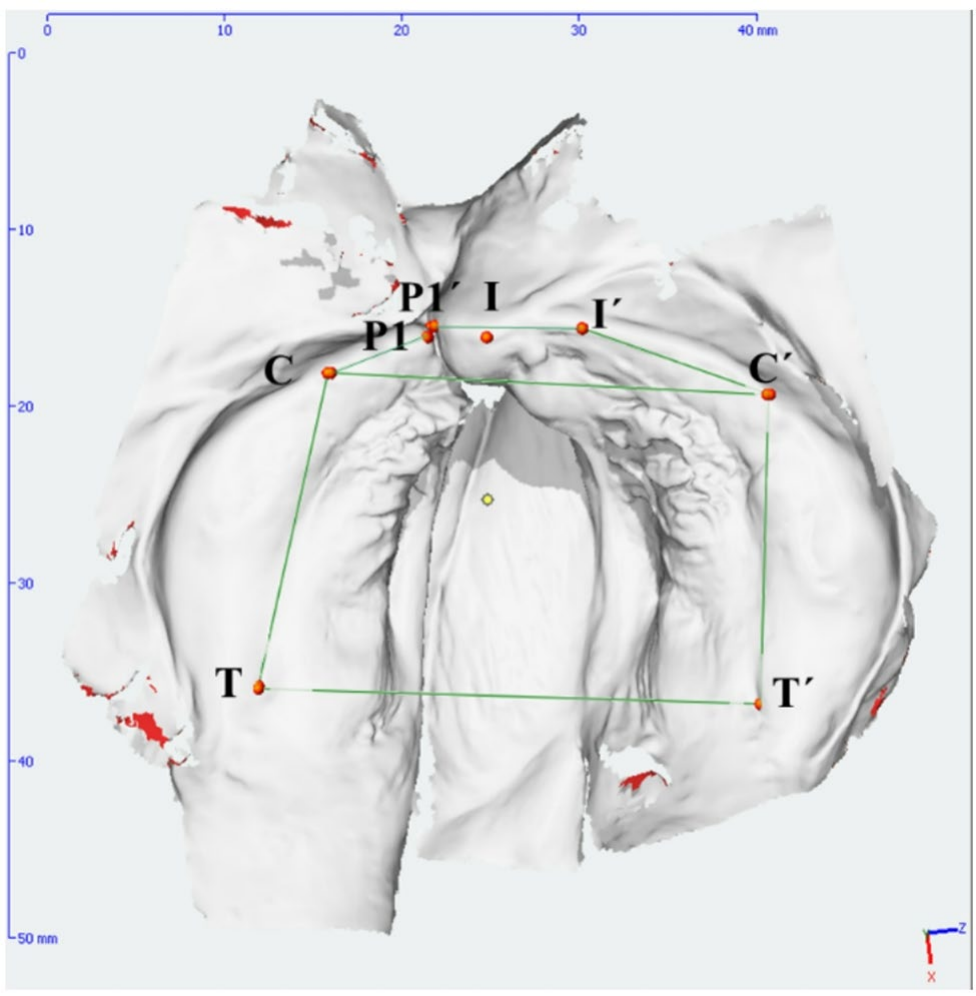
3D model with markings of the reference points and measured distances

Although this method produces 3D models of the maxilla, the subsequent model analysis was still performed in two dimensions using the reference points and lines to ensure comparability with previous studies. For the conventional method, various reference points (Fig. 1; Table 2) were manually defined and marked on the 3D models. Canine distance and jaw segment length were calculated using OnyxCeph3™ software (https://onyxceph.eu/; Image Instruments GmbH, Chemnitz, Germany; Fig. 1; Table 3).

**Table 2:** Reference points and their definition

Description	Reference Point	Definition
T	Tuber maxillae left	Transition of the alveolar ridge into the contour of the maxillary tuberosity
T´	Tuber maxillae right	
P1/P1´	border of the right cleft	
P2/P2´	border of the left cleft	
C	Left first canine point	Connection of the lateral sulcus with the alveolar ridge (dental papilla between canine and first deciduous molar)
C´	Right first canine point	
I	Left incisal point	Line connecting the incisal papilla and the labial frenulum (refers to the interdental papilla of the central incisors)
I´	Right incisal point	

**Table 3:** Outcome parameter definitions

Outcome parameter	Definition
C-C´	intercanine distance – transversal distance between the eruption points of the left and right canine
T-C-P2/P1´-C´-T´	jaw segment length of the cleft side
P2´-I´-C´-T´/ P1-I-C-T	jaw segment length of the non-cleft side

**Fig. 2:**
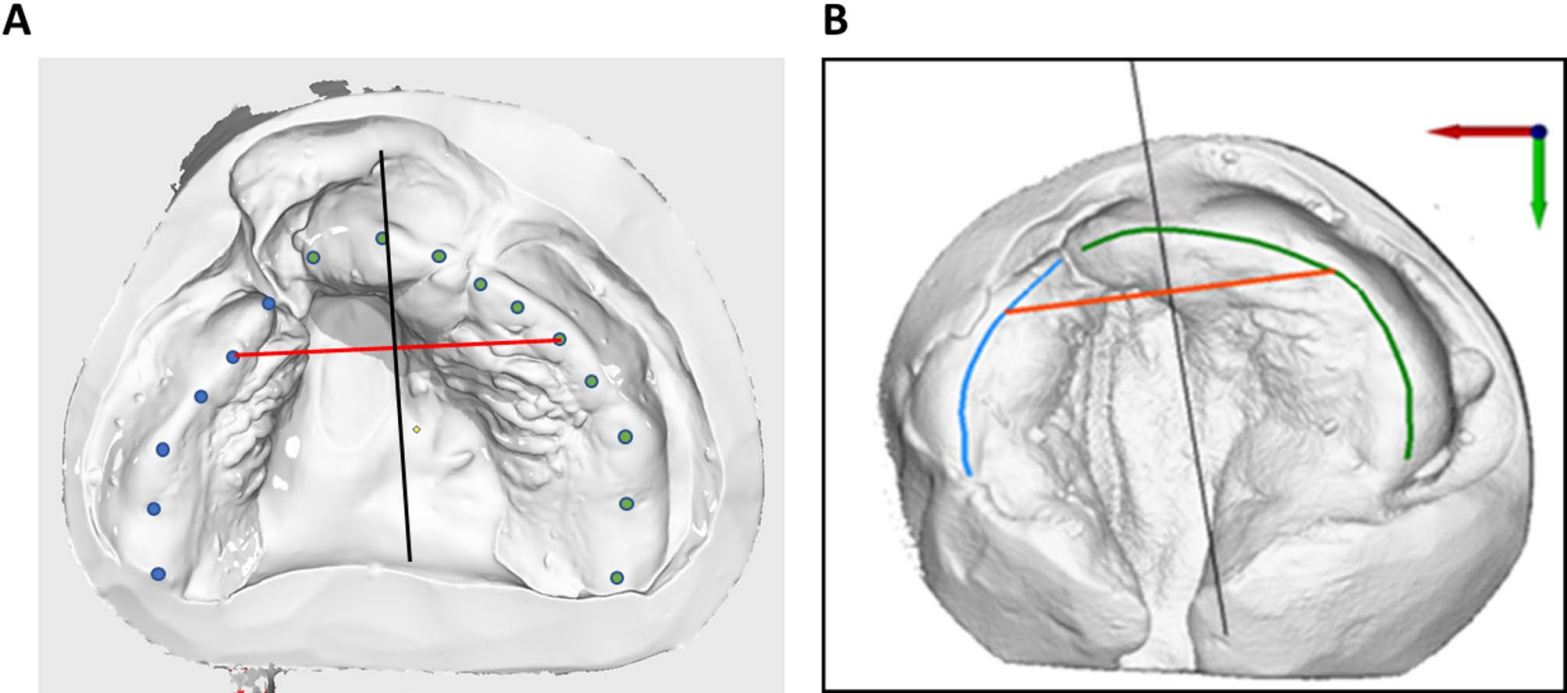
Example of the evaluation of digitized plaster models. (**A)** Marking of the alveolar ridges using points on the alveolar edge crest (blue = cleft side; green lines = non-cleft side) (**B**) Automatically drawn polylines based on the marked points. The red line corresponds to the transversal connection of the eruption points of the primary canines (canine distance). The black vertical line is drawn perpendicular to the canine distance line through its center point

For the semi-automatic evaluation, we used the software developed by Dr.-Ing. S. Holtzhausen the Chair of Virtual Product Development, Working Group Reverse Engineering at the TU Dresden (KTC-stl-viewer; www.re-dresden.de; Windows 10; programming language: C#). The larger and smaller split segments were marked manually with 5–10 points on the alveolar ridge, outlining the entire palatal margin and ending at the rearmost point of the tuber, as described above [10, 26, 27]. Where teeth were present, these points were adjusted to align with the gingival margins at the palatal side of the teeth (Fig. 2A).

The manually marked points of the alveolar ridge were then automatically connected by the software to form polylines and projected onto a plane, in our case the occlusion plane, in order to describe the alveolar ridge using a mathematical, two-dimensional function (Fig. 2B). To determine the position of the canines (Table 2), a straight line (red line) was drawn between them, and a vertical line perpendicular to this connecting line (black line) was used to assess jaw symmetry (Fig. 2).

**Fig. 3:**
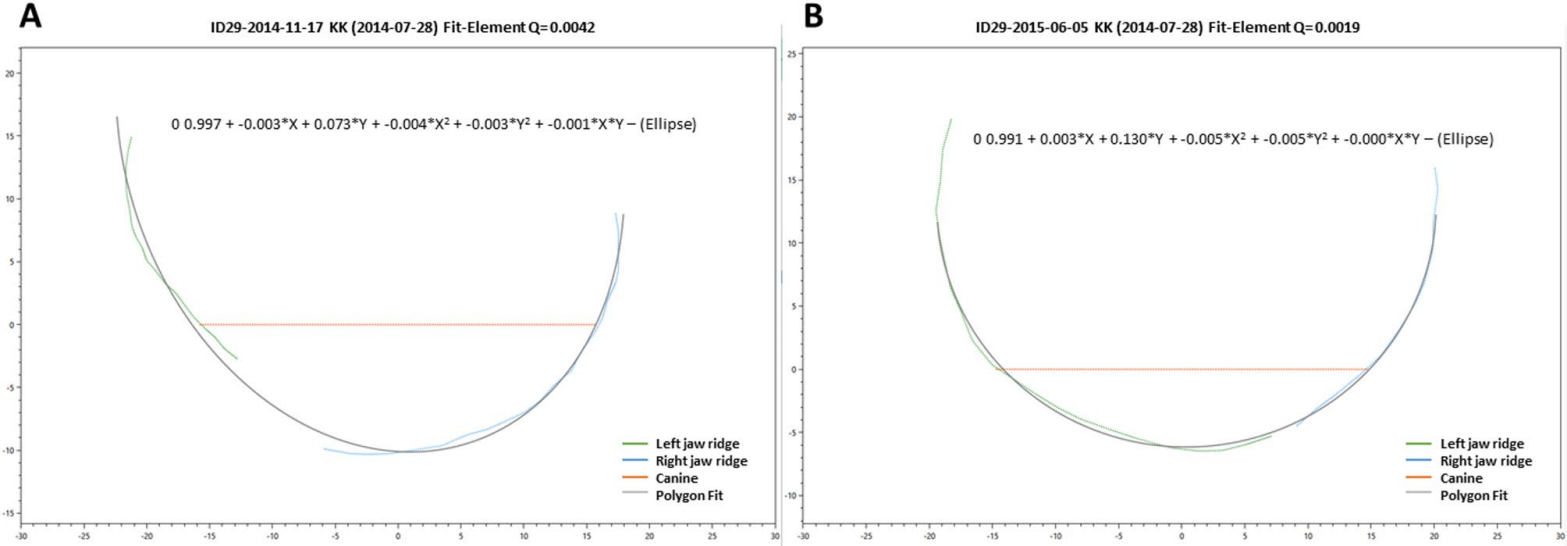
Measurement of the deviation of the course of the jaw ridges (blue and green lines) from the ideal curve (gray line/ellipse) on the upper jaw models (**A**) before lip plastic (T1) and (**B**) before palate closure (T2)

A second-order curve was fitted to all points of the two individual curves of the cleft and non-cleft segments using the least squares method, and the average deviation of the polylines from the second-order curve function was determined (Fig. 3). The second order curve is assumed to adequately describe the continuity of the two halves of the jaw. A polynomial was used for this curve and all parameters G0 to G5 were determined during fitting:

$$0=G0\mathit\;+\mathit\;G1\cdot\mathrm x\;+\;G2\cdot\mathrm y\;+\;G3\cdot\mathrm x^2\;+\;G4\cdot\mathrm y^2\;+\;G5\cdot\mathrm x\cdot\mathrm y$$

Various simple curves (straight lines, parabolas, paraboloids, and ellipses) can be plotted. In this study, we refer to an ellipse because the deviation of the alveolar ridge points from the approximate curve is crucial for assessing the continuity of the dental arch (Fig. 3). The complete workflow in the software KTC-stl-viewer is shown in the additional PowerPoint presentation attached (supplemented materials).

### Statistical analysis

Three independent measurements were taken by two independent examiners to ensure accuracy and reproducibility. A time interval of at least one week was maintained between repeated measurements within the measuring procedure. To estimate the intra-observer variation of the measurement procedure, the standard deviation was calculated for three repeated measurements, and then averaged across all positions and patients.

The deviation of the individual software-generated elliptical polylines from the ideal ellipse was quantified using the Q coefficient, which measures the agreement between the traced curve and the mathematically adjusted ellipse. This quantification was performed on all models at times T1 and T2. The normal distribution of the data was first checked, and then Q coefficients at times T1 and T2 were compared and statistically evaluated. Statistical analysis of the data was carried out using SPSS Statistics 29.0 software (IBM, USA). If the curvature of the jaw changes, the distances to the polynomials increase. The greater the Q coefficient, the greater the deviation of the measuring points from the polynomials.

## Results

### Intra-observer variability

The intra-individual deviations between the three measurements performed by both examiners were determined for the intercanine distance. Linear measurement using OnyxCeph yielded an intra-observer variability of 0.58 ± 0.33 mm, whereas a value of 0.34 ± 0.15 mm was determined when using the self-made bespoke software application (SMP).

### 2D conventional analysis

Using conventional linear methods, we marked the jaw segments of the cleft and non-cleft sides (Table 2 and 3) in the OnyxCeph program and measured the length of the entire segment (Table 4).

**Fig. 4:**
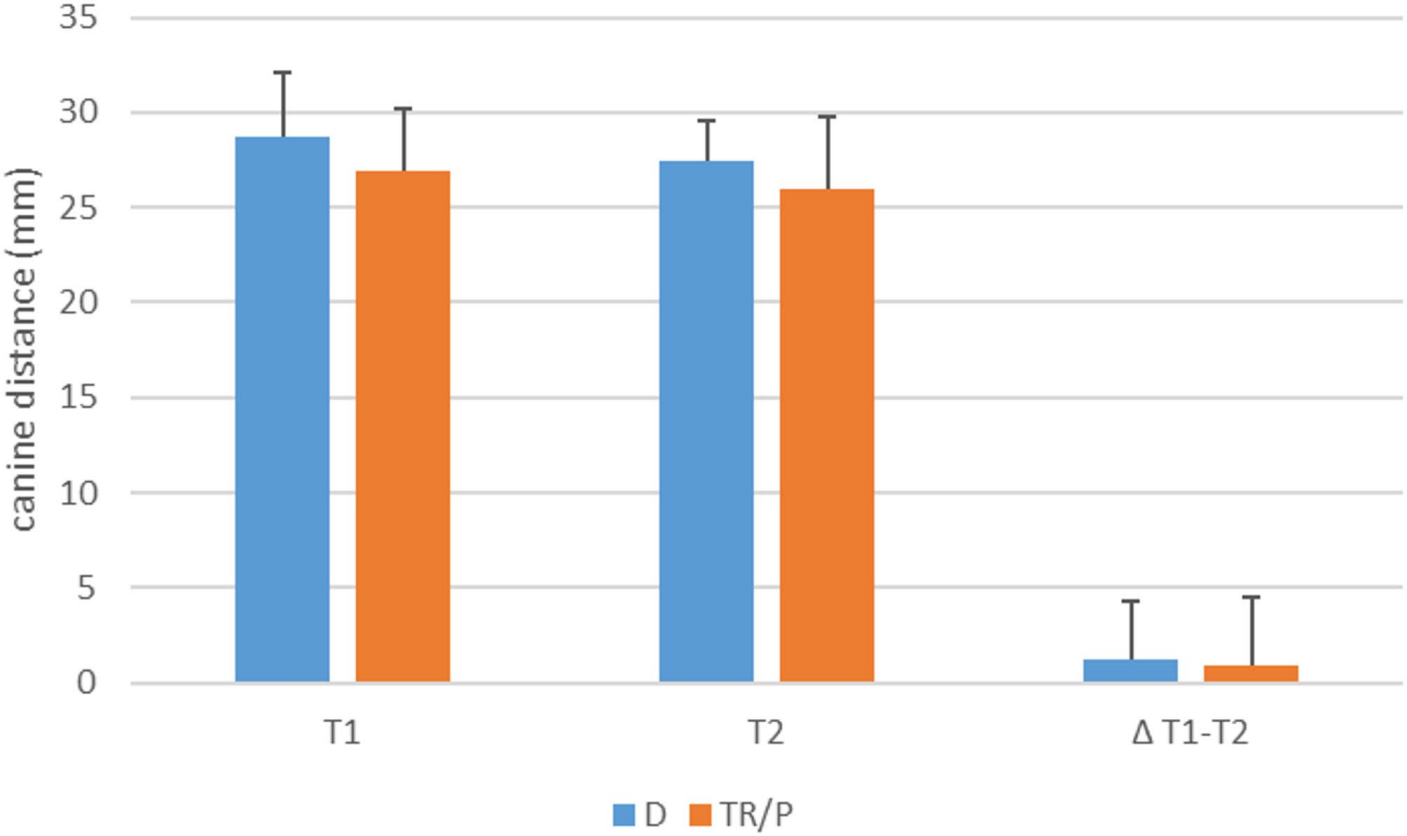
Measurement of the intercanine distance at time T1 and T2 and calculation of the difference between T1-T2. The mean ± standard deviation for the respective study groups were shown. D = Delaire procedure; TR/P = Tennison-Randall/Pfeifer procedure

Secondly, we measured the canine distance at time T1 and T2 and calculated the difference between T1-T2. Using the conventional method, no differences were found in the measured canine distance before and after lip closure with the two different surgical techniques (Fig. 4).

### Self-made bespoke software application (SMP)

#### Comparison with the 2D-conventional analysis

We performed the semi-automated measurements using a self-developed customized software application (SMP) tailored to the specific measurement requirements of this study. The results of the alveolar ridge lengths were first compared with those of the OnyxCeph analysis. Both measurements were shown to be statistically significantly different on both the cleft and non-cleft sides. The measured alveolar ridge lengths were longer with the SMP than with the OnyxCeph measurement (Table 4).

**Table 4:** Comparison of alveolar ridge lengths (in mm) between SMP and onyxceph evaluation

	Cleft Side (OnyxCeph) (Mean ± SD)	Cleft Side (SMP) (Mean ± SD)	Non-Cleft Side (OnyxCeph) (Mean ± SD)	Non-Cleft Side (SMP) (Mean ± SD)
	24.61 ± 7.24	28.77 ± 10.38	34.5 ± 7.81	41.45 ± 8.79
*P* value (Onyx vs. SMP)	0.0329		0.033	

#### 3D analysis

**Fig. 5:**
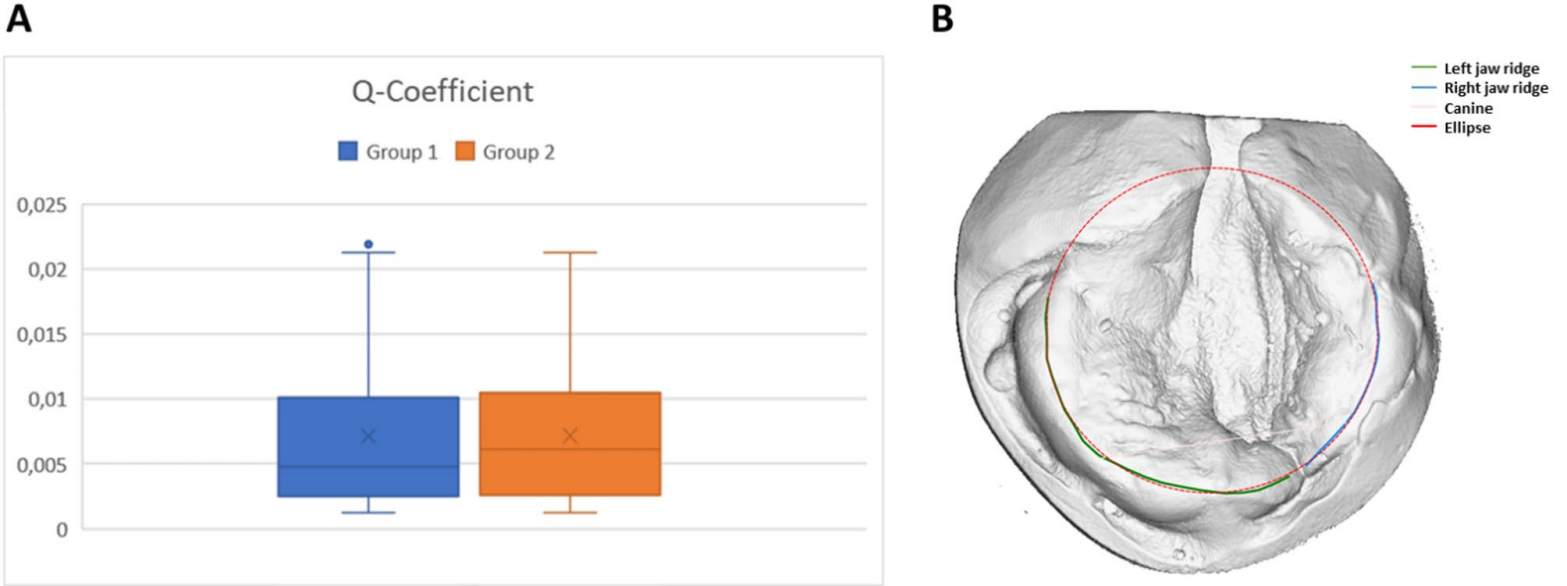
**(A)** Boxplot of the Q coefficient calculated between time points T1 and T2 in both groups and (**B**) illustration of the minimal difference between the ideal ellipse (red) and the manually marked ellipses (green and blue line). D = Delaire procedure; TR/P = Tennison-Randall/Pfeifer procedure

By measuring the deviations of the distance of the jaw segments from the ideal ellipse design, we found a statistically significant difference in the Q coefficient between the two surgical procedures for the difference between time points T1 and T2 in the Wilcoxon rank sum test with continuity correction (W = 962, p-value = 0.001393; Fig. 5).

## Discussion

In recent decades, many attempts have been made to measure and compare changes in the facial surface using facial reconstruction. Many publications have proposed methods for analyzing shape in two dimensions. These analyses depend on reference points being easily and unambiguously identifiable [28–30]. This is not the case with slightly curved surfaces, such as most of the human face or the alveolar ridge. When measuring, it is important to examine ‘the curved surface that carries the reference points, and not the reference points themselves’. Firstly, as Coombes described in 1991, mathematical methods can be used to circumvent this problem [30]. Secondly, the problem can be avoided by using 3D imaging. 3D imaging has become an indispensable tool in modern orthodontics, offering improved diagnostic capabilities and treatment planning precision [31]. Its benefits include early detection of problems and improved surgical planning, ultimately leading to more efficient and effective orthodontic and surgical care [32]. It has also been shown in the meantime that the 2D analysis of dental plaster casts is not sufficiently informative, especially not for the three-dimensional changes in cleft palates. Furthermore, Pouliezou points out that in more complex cases, where prediction and accurate performance are important, a 3D model is needed [33]. Undoubtedly, UCLP patients fall into this category.

This study presents a method to visualize and quantify changes in the growth and symmetry of the maxilla after the primary closure of lip cleft using two surgical techniques. In our study we used both a semi-automated mathematical analyzes using 3D imaging and the 2D measurement. While there were significant differences in the measurements of the length of the alveolar ridges taken with OnyxCeph3™ and our custom program (SMP), no difference was found in the linear measurements of the distance between canines. This consistency in canine distance suggests that certain linear measurements may be less affected by differences in measurement methodology. This may indicate that these methods may lack the sensitivity to detect certain types of changes, which may be a limitation of traditional measurement techniques as described above. However, for more complex segment distances, the different linear algorithms and reference points used by each program may lead to discrepancies. This highlights a potential limitation of linear measurement approaches, especially when comparing results between different software programs, as different methodologies may affect precision and accuracy. The discrepancy between 2D and 3D analyses could recently also be shown for the accuracy between intraoral scans and digitized plaster models in cleft patients [34]. The 2D measurement gives the impression that the accuracy of intraoral scans is not as good as that of conventional plaster casts in cleft patients. However, the 3D measurements showed very good agreement between the two impression methods [34]. Only the semi-automatic analysis led to a viable, easily reproducible and repeatable measurement of plaster casts with the subsequent possibility of more complex statistical analysis. In the context of cleft care and orthodontics, assessing maxillary morphology is particularly challenging due to the complex and asymmetric growth patterns that are typical for UCLP patients [35–37]. Therefore, a combination of methods is often required to achieve clinically meaningful evaluation. Our approach provides a structured way to integrate traditional anatomical landmarks with geometric curve modeling. While the curve-fitting approach itself is mathematically established, the novelty of our method lies in its clinical application: integrating anatomical landmark-based orientation, semi-automated ridge tracing, and symmetry evaluation through an ellipse fitting process specifically tailored to the assessment of maxillary morphology in UCLP patients. To our knowledge, no prior study has combined these elements into a reproducible workflow using 3D models in this patient population. To validate the method, the dimensions of the maxillary arch were analyzed and compared in children with UCLP who had undergone Delaire or Tennison-Randall/Pfeifer surgery prior to lip and palate closure. We found a statistically significant difference in the Q coefficient between the two surgical procedures. Whether this difference in the shape of the dental arch found early in maxilla development will persist and have any impact on the extent of orthodontic treatment and the need for surgical corrections become apparent in the years to come needs to be seen throughout growth until adulthood. Studies have shown differences between surgical procedures, indicating that the Tennison procedure requires a shorter orthodontic treatment period and is associated with fewer tooth injuries compared to the Millard technique [11]. We need to monitor these children in order to further elucidate differences once they have reached adulthood. However, this will only be possible if the monitoring methods are simple and easy to evaluate. Importantly, while intraoral scanning and fully digital workflows are becoming more common, they are not yet universally implemented in clinical or archival practice. It may take decades before large, consistently scanned 3D datasets are available across institutions. Therefore, having a method that can be retroactively applied to conventional plaster models—such as the one presented here—is essential for long-term and comparative cleft research.

Children with cleft palate often have a collapsed maxillary arch, which can be a challenge for orthodontic treatment [14, 15]. The maxillary arch is the upper jaw bone that supports the upper teeth, and its collapse can result in a narrow palate and malocclusion, or misalignment of the teeth [38]. Correcting collapsed maxillary segments in children with cleft palate is critical for both function and aesthetics. A narrow maxillary arch can cause breathing, speech and feeding difficulties, as well as affecting facial appearance. Effective orthodontic therapy requires a comprehensive approach to restore function and improve aesthetics. A good understanding of the effects of primary surgery and the correct choice of surgical treatment is essential for the rehabilitation of cleft patients and forms the basis for appropriate orthodontic treatment with optimal results. There has already been evidence of changes in the maxillary arches of children with UCLP and cleft palate as a result of primary surgery [39].

Due to the limited follow-up period of this study, which examines the growth trend only within the first-year post-surgery, gender differences were not considered. The same was found in the study of Sakoda et al. [39]. Research indicates that gender differences in the first year of life are mainly related to weight and body assessment [40]. Further studies are needed to evaluate the long-term outcomes of different surgical techniques on the maxillary arch morphology in infants with UCLP.

## Conclusion

The results suggest that the semi-automated method could provide a more accurate assessment of surgical outcomes, which may inform adjustments to surgical techniques for cleft lip and palate repair. Additionally, we validated the method by comparing it to conventional linear analysis (OnyxCeph3™) and assessed intra-observer variability, which showed high measurement consistency. This positions our method as a practical and semi-automated tool for evaluating the morphological impact of surgical techniques in cleft care, even on existing plaster model. This bridges the gap between traditional documentation and modern 3D analysis.

## Supplementary Information

Supplementary Material 1.

## Data Availability

The datasets used and/or analysed during the current study are available from the corresponding author on reasonable request.

## Abbreviations

2D: Two-dimensional
3D: Three-dimensional
UCLP: Unilateral cleft lip and palate
SMP: Self-developed customized software application
D: Delaire
TR/P: Tennison-Randall / Pfeifer
STL: Standard tessellation language
