# Supplementary material for: Influence of the surgical method used for lip closure on the shape of the maxillary arch: a retrospective 3D analysis of plaster models
Source: Head Face Med. 2025 Oct 28;21:72. doi: 10.1186/s13005-025-00550-5 (PMC12560360; doi:10.1186/s13005-025-00550-5)
Supplement: Supplementary file 1 — Supplementary Material 1. [file 13005_2025_550_MOESM1_ESM.pdf]

The steps to be carried out in the softfware are marked in red.

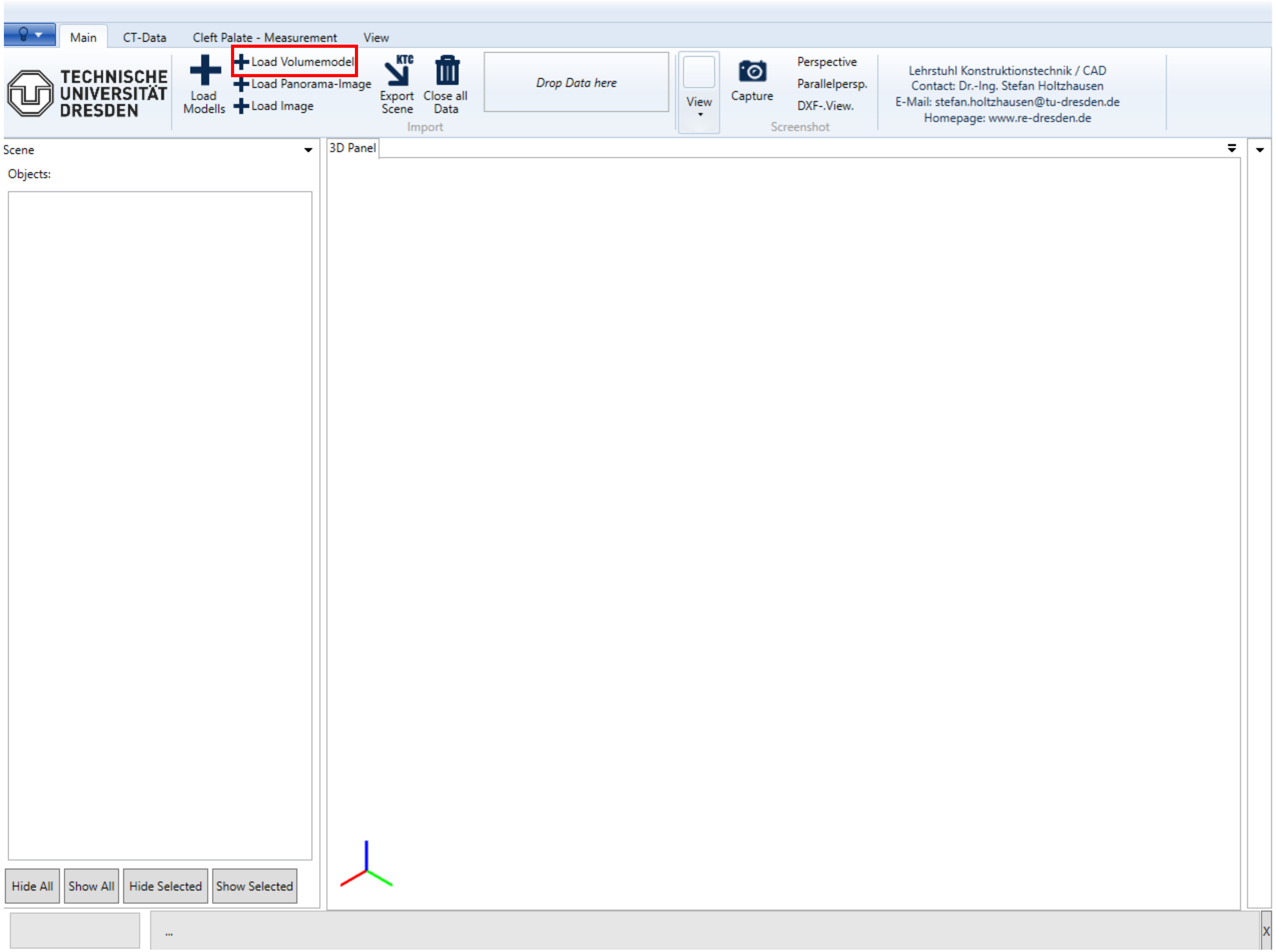

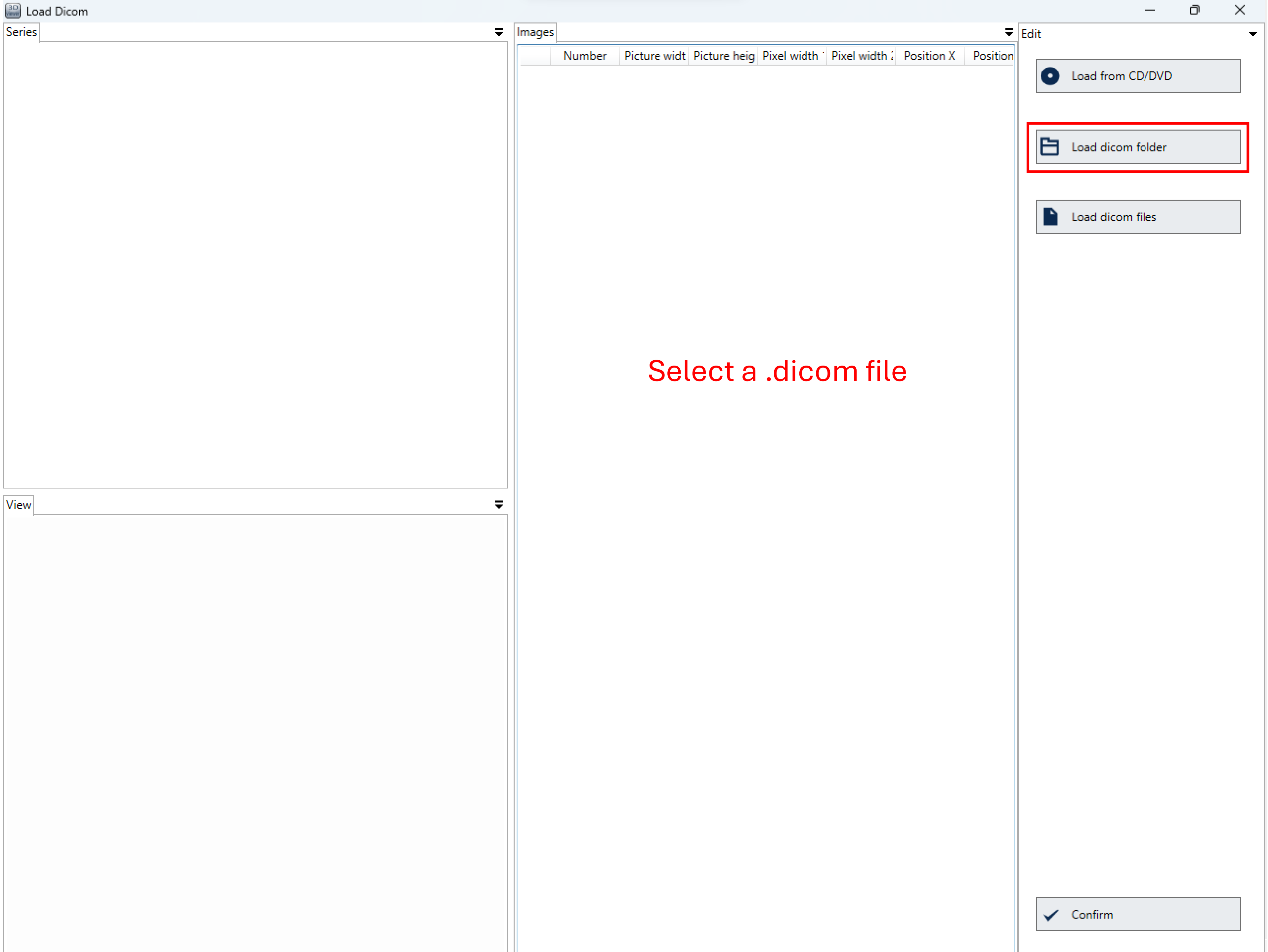
Load Dicom
Series
Images
Edit
Number
Picture widt
Picture heig
Pixel width
Pixel width
Position X
Position
Load from CD/DVD
Load dicom folder
Load dicom files
Select a .dicom file
View
Confirm

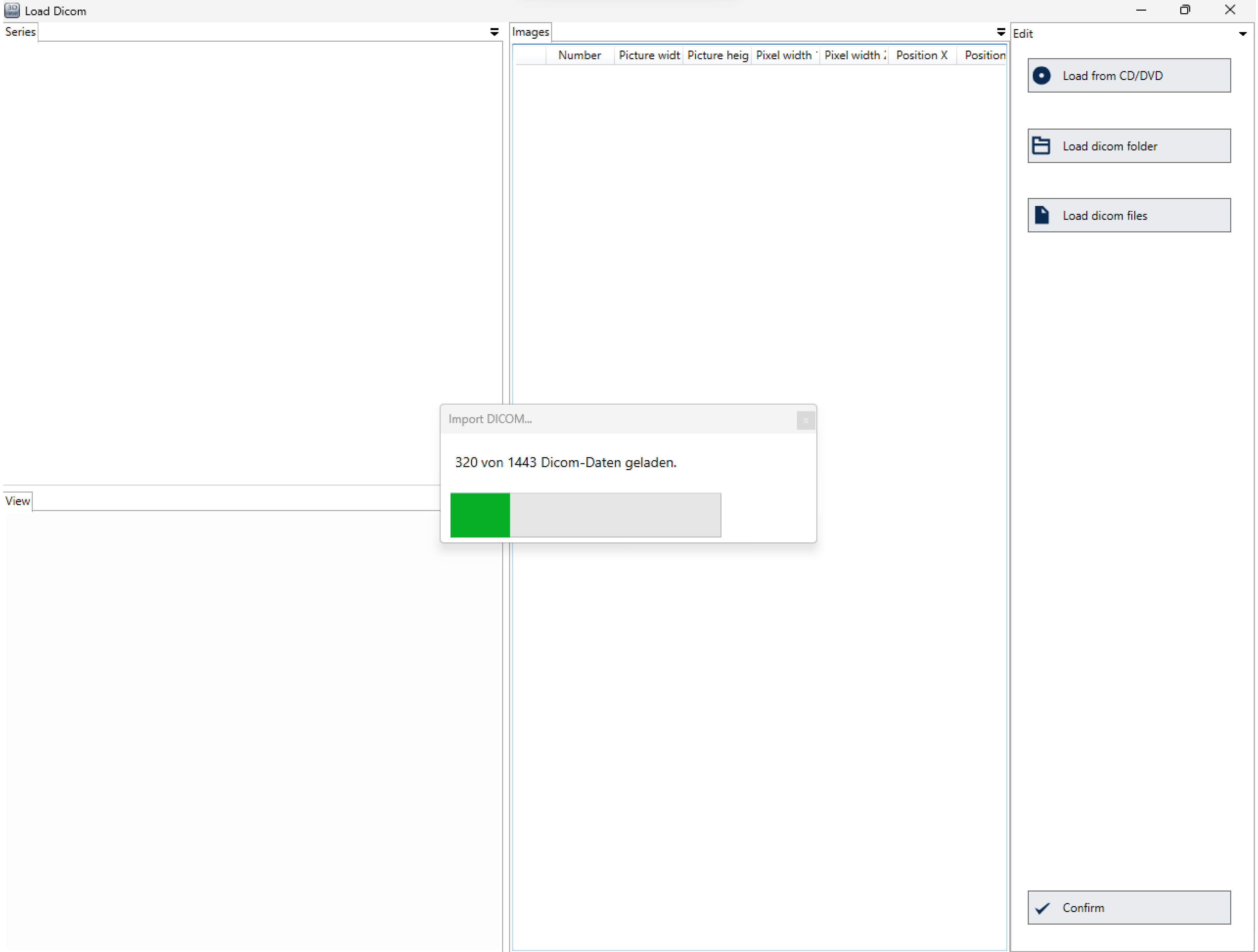
Load Dicom
Series
Images
Number
Picture widt
Picture heig
Pixel width
Pixel width
Position X
Position
Edit
Load from CD/DVD
Load dicom folder
Load dicom files
Import DICOM...
320 von 1443 Dicom-Daten geladen.
View
Confirm

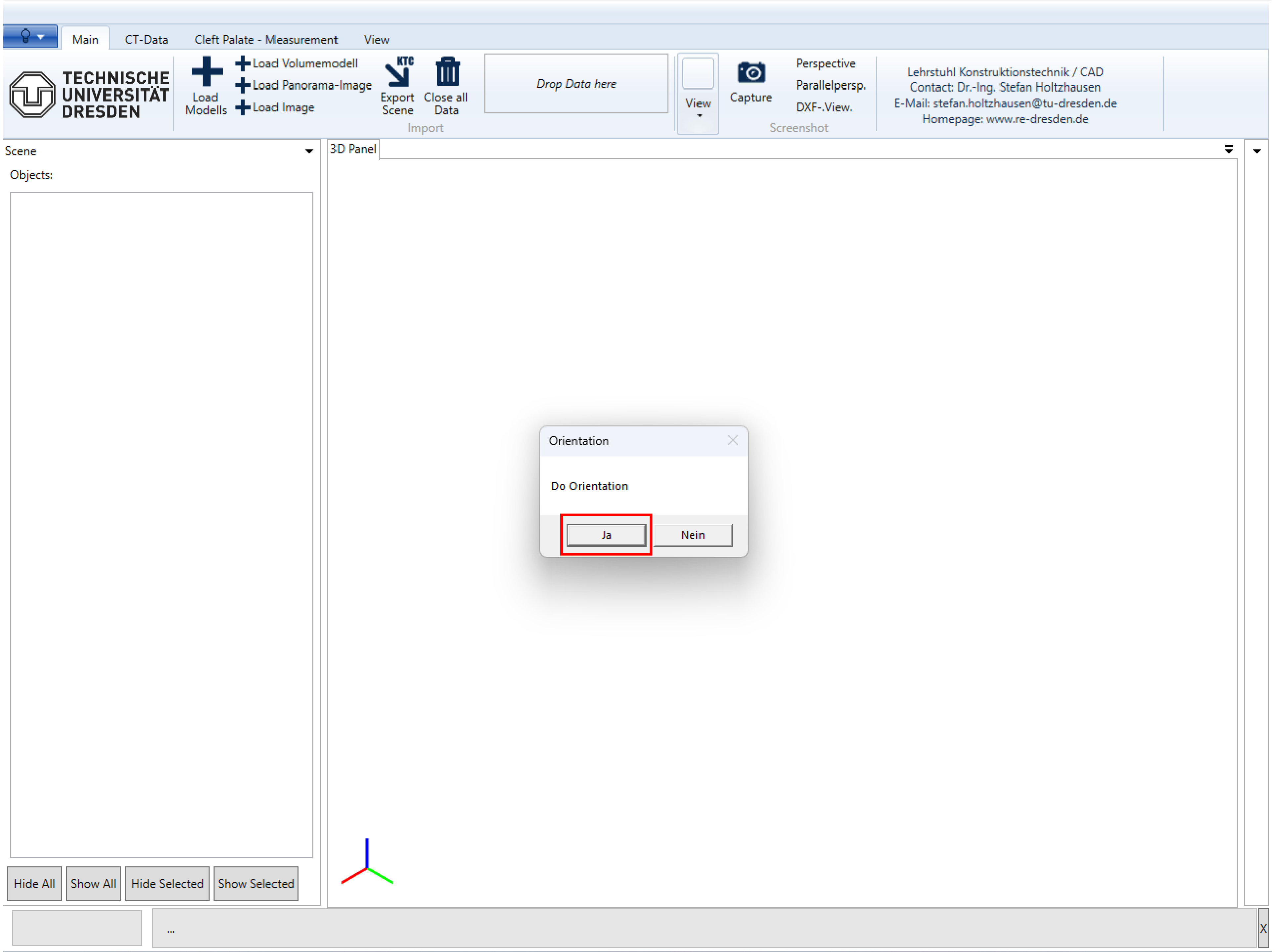
Main
CT-Data
Cleft Palate - Measurement
View
TECHNISCHE UNIVERSITÄT DRESDEN
Load Modells
Load Volumemodell
Load Panorama-Image
Load Image
Export Scene
Close all Data
Import
Drop Data here
View
Capture
Perspective
Parallelpersp.
DXF-.View.
Screenshot
Lehrstuhl Konstruktionstechnik / CAD
Contact: Dr.-Ing. Stefan Holtzhausen
E-Mail: stefan.holtzhausen@tu-dresden.de
Homepage: www.re-dresden.de
Scene
Objects:
3D Panel
Orientation
Do Orientation
Ja
Nein
Hide All
Show All
Hide Selected
Show Selected

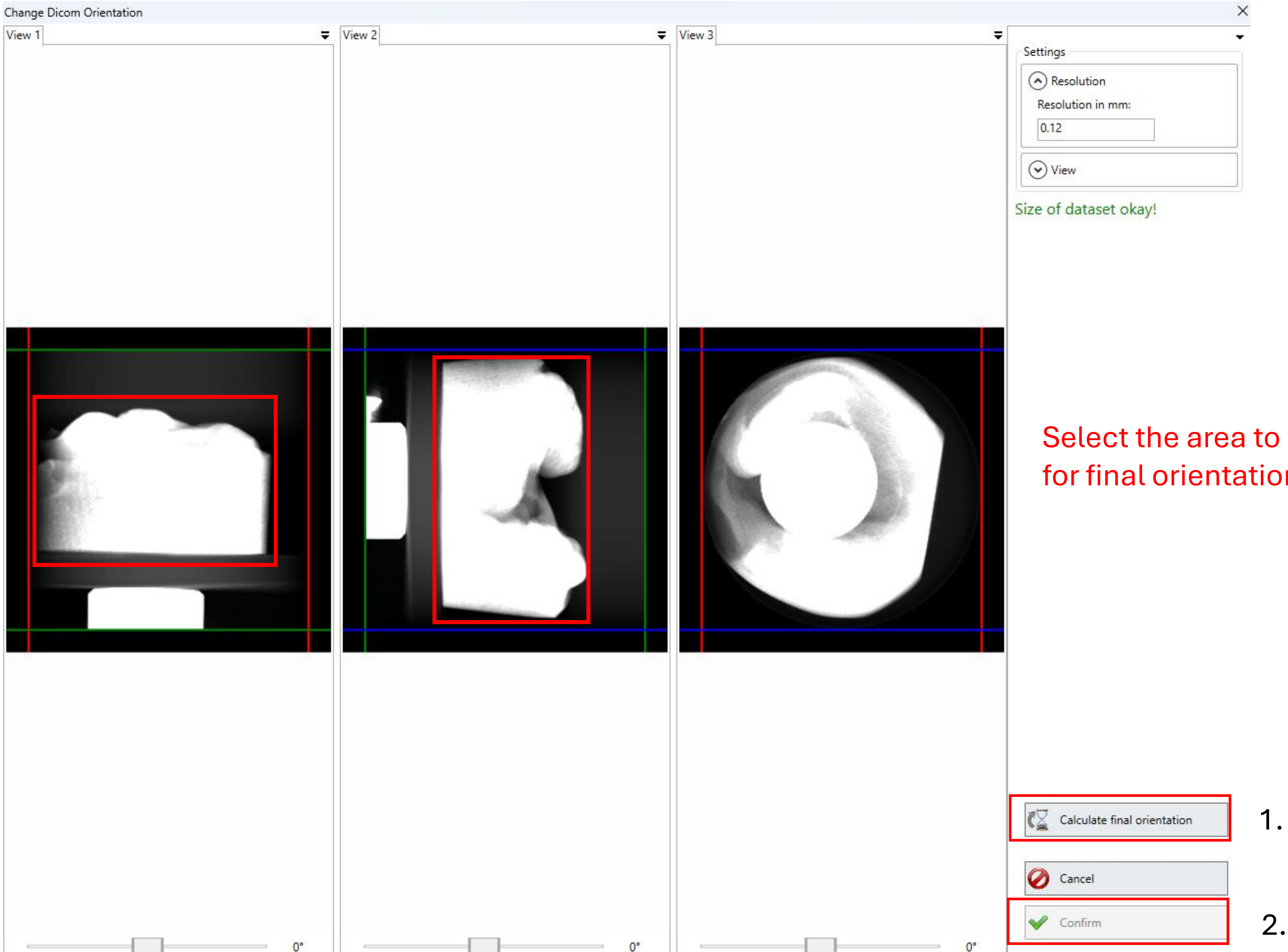
Change Dicom Orientation
View 1
View 2
View 3
Settings
Resolution
Resolution in mm:
0.12
View
Size of dataset okay!
Select the area to be used for final orientation.
Calculate final orientation
1.
Cancel
Confirm
2.
0°
0°
0°

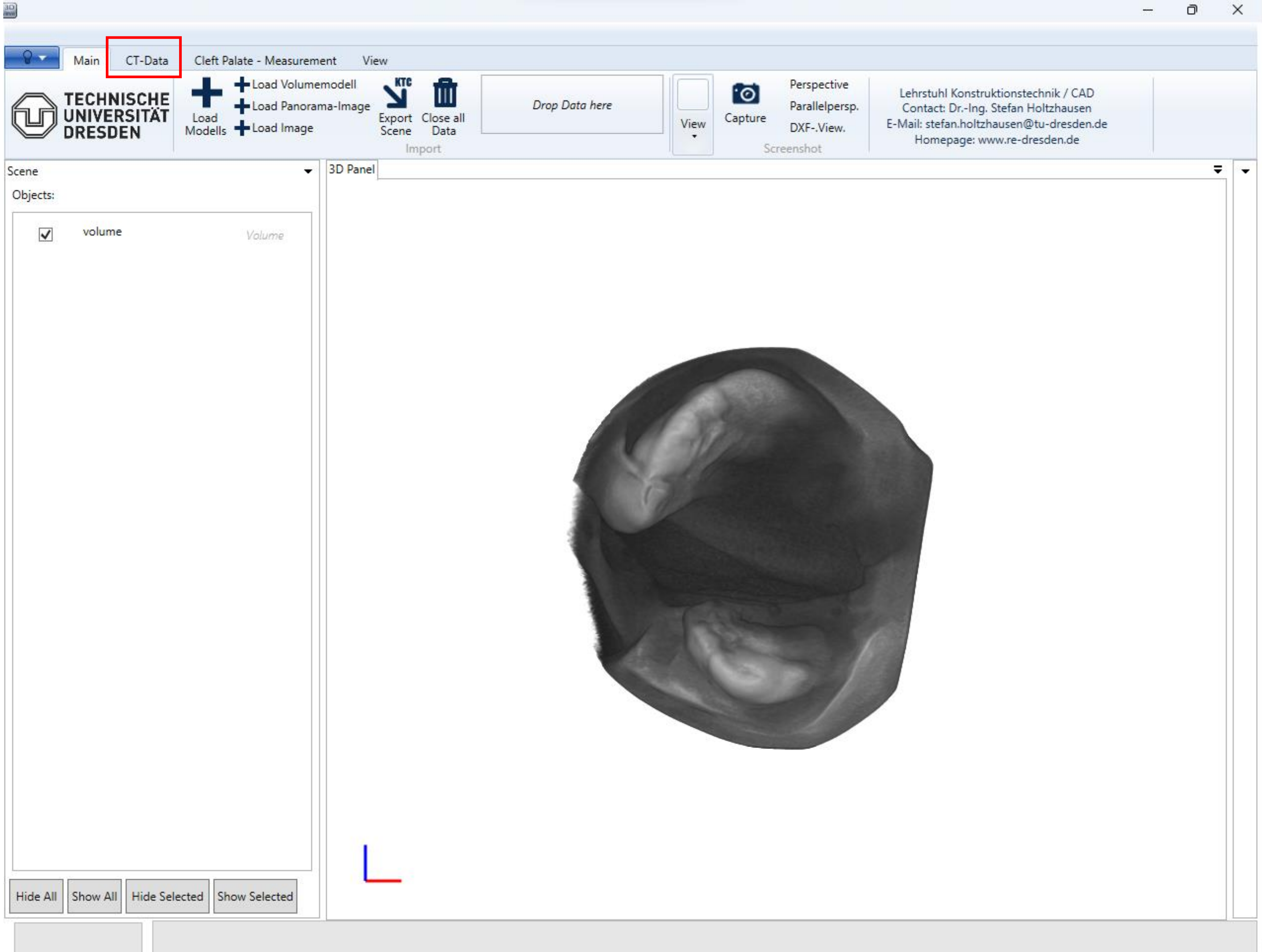
Main
CT-Data
Cleft Palate - Measurement
View
TECHNISCHE UNIVERSITÄT DRESDEN
Load Modells
Load Volumemodell
Load Panorama-Image
Load Image
Export Scene
Close all Data
Import
Drop Data here
View
Capture
Perspective
Parallelpersp.
DXF-.View.
Screenshot
Lehrstuhl Konstruktionstechnik / CAD
Contact: Dr.-Ing. Stefan Holtzhausen
E-Mail: stefan.holtzhausen@tu-dresden.de
Homepage: www.re-dresden.de
Scene
Objects:
volume
Volume
3D Panel
Hide All
Show All
Hide Selected
Show Selected

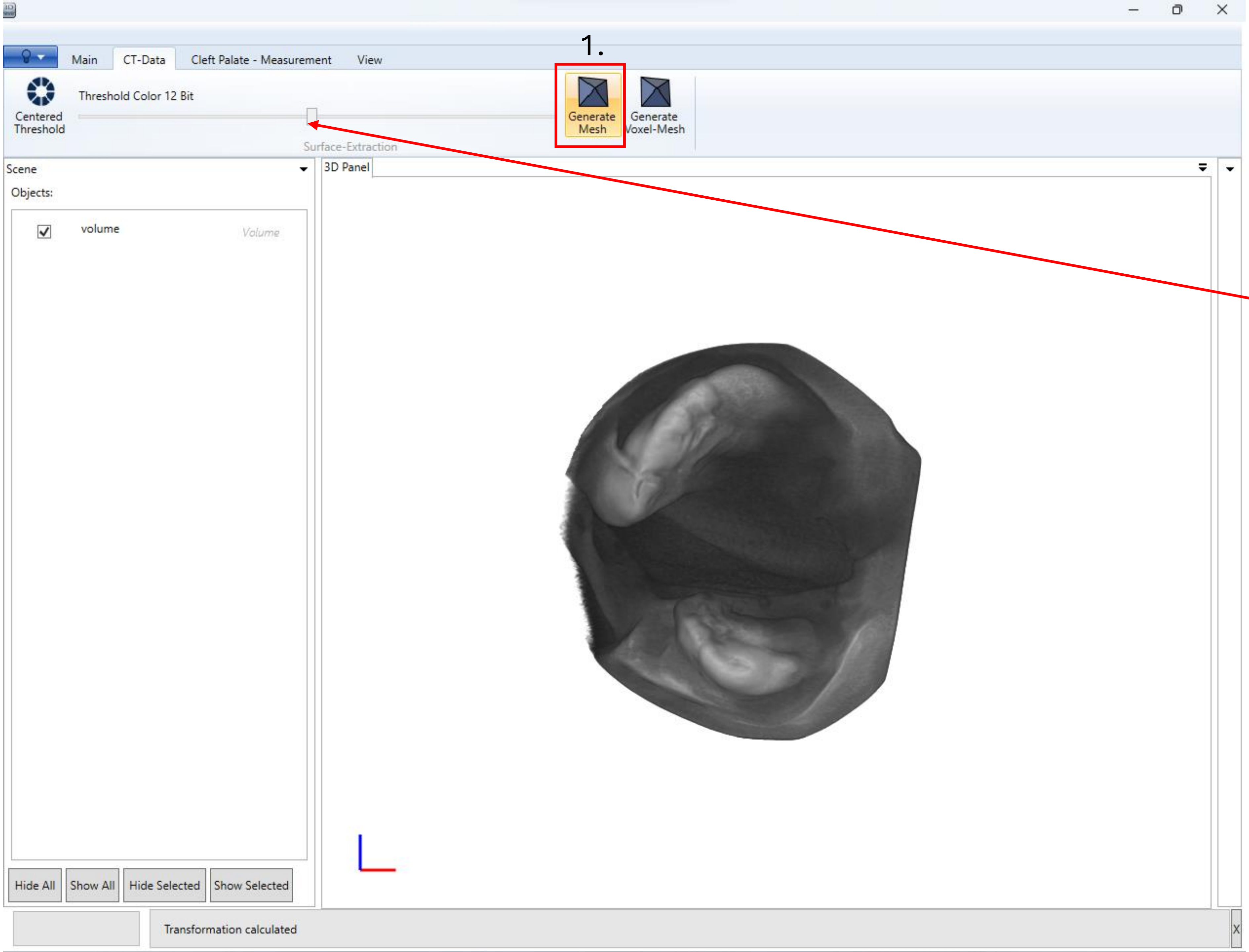

2.

After creating the mesh, the threshold color can be changed.

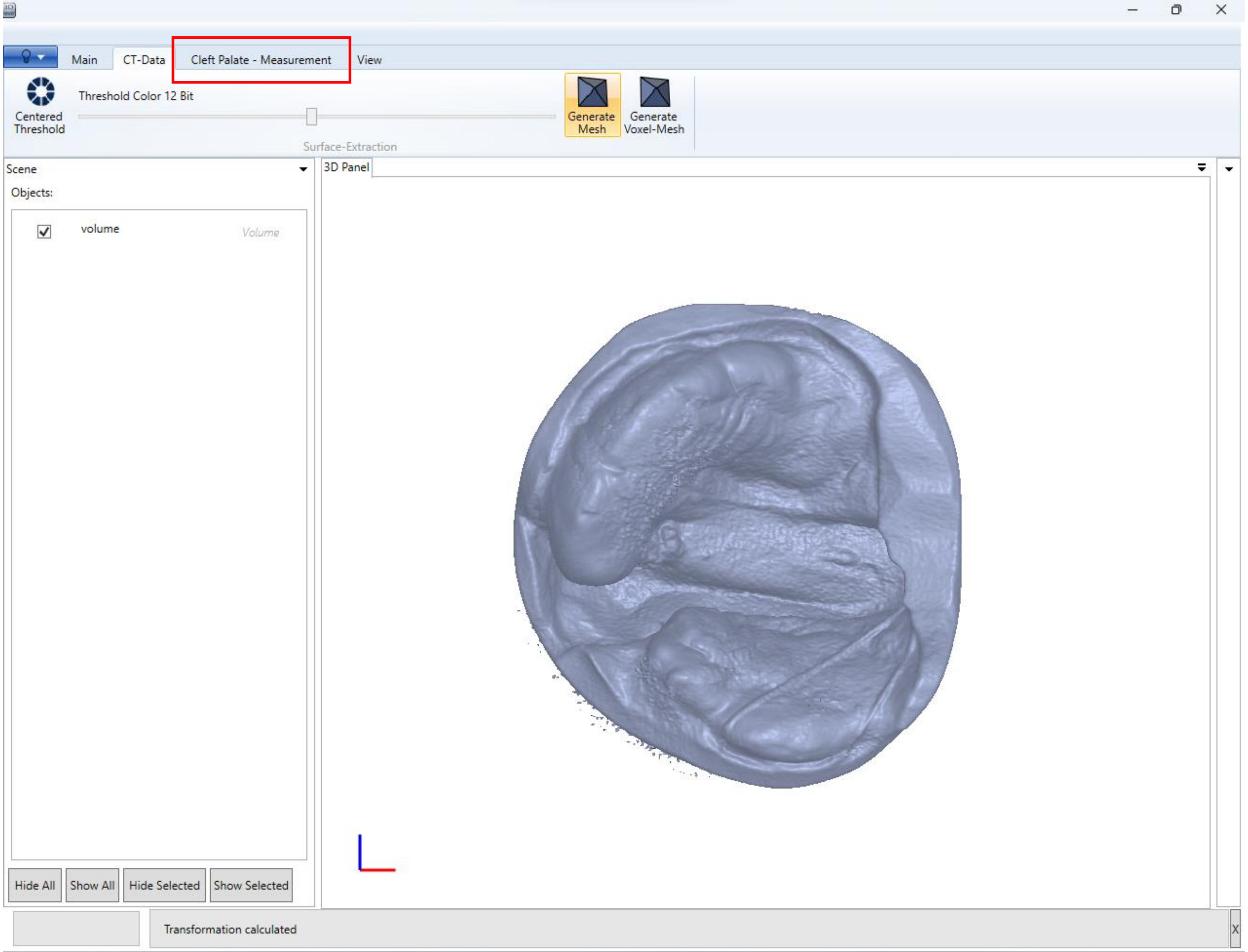
Main
CT-Data
Cleft Palate - Measurement
View
Centered Threshold
Threshold Color 12 Bit
Generate Mesh
Generate Voxel-Mesh
Surface-Extraction
Scene
Objects:
volume
Volume
3D Panel
Hide All
Show All
Hide Selected
Show Selected
Transformation calculated

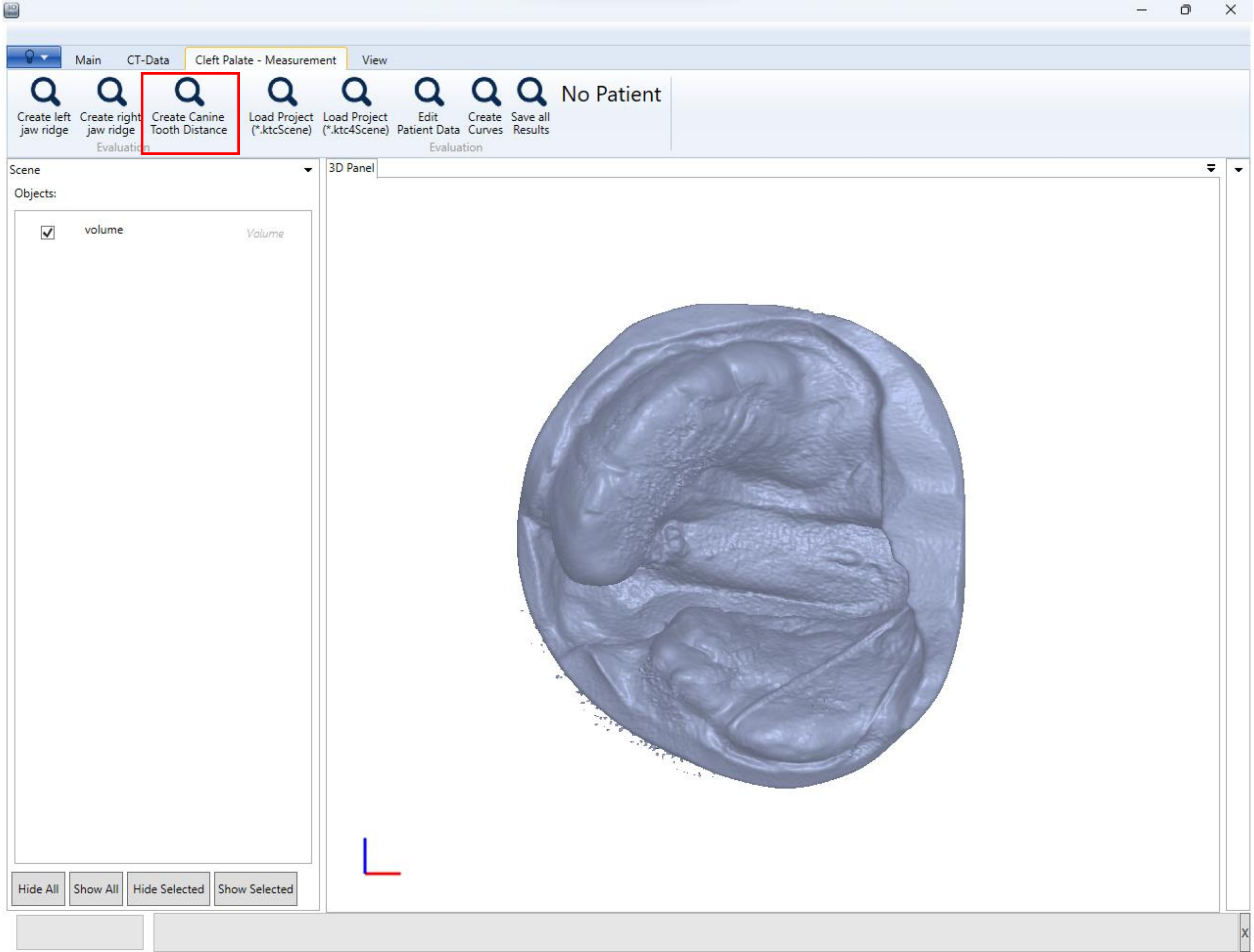
Main
CT-Data
Cleft Palate - Measurement
View
Create left jaw ridge
Create right jaw ridge
Create Canine Tooth Distance
Evaluation
Load Project (*.ktcScene)
Load Project (*.ktc4Scene)
Edit Patient Data
Create Curves
Save all Results
Evaluation
No Patient
Scene
Objects:
volume
Volume
Hide All
Show All
Hide Selected
Show Selected
3D Panel

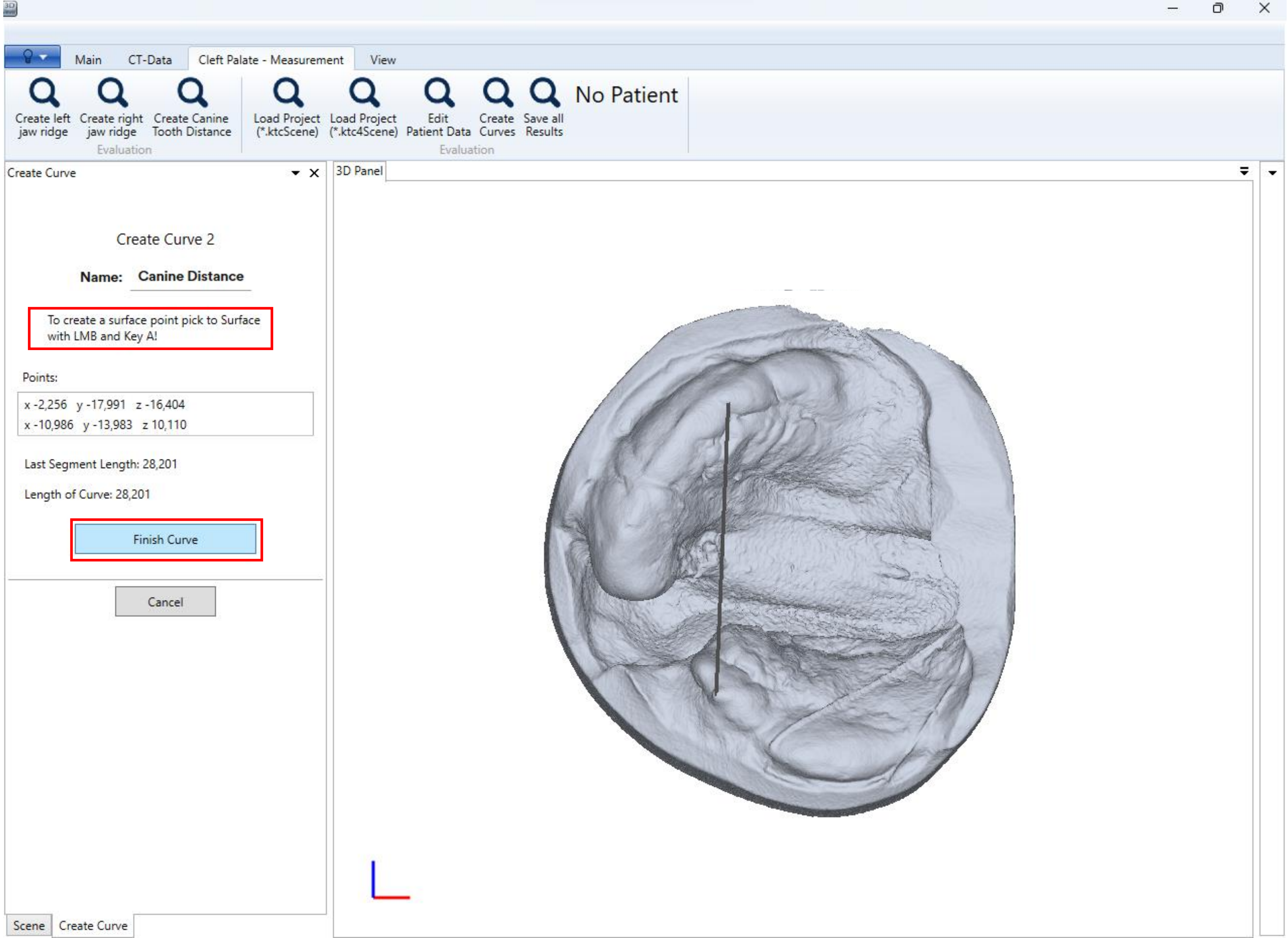
Main
CT-Data
Cleft Palate - Measurement
View
Create left jaw ridge
Create right jaw ridge
Create Canine Tooth Distance
Evaluation
Load Project (*.ktcScene)
Load Project (*.ktc4Scene)
Edit Patient Data
Create Curves
Save all Results
Evaluation
No Patient
Create Curve
3D Panel
Create Curve 2
Name: Canine Distance
To create a surface point pick to Surface with LMB and Key A!
Points:
x -2,256 y -17,991 z -16,404
x -10,986 y -13,983 z 10,110
Last Segment Length: 28,201
Length of Curve: 28,201
Finish Curve
Cancel
Scene
Create Curve

1.

2.

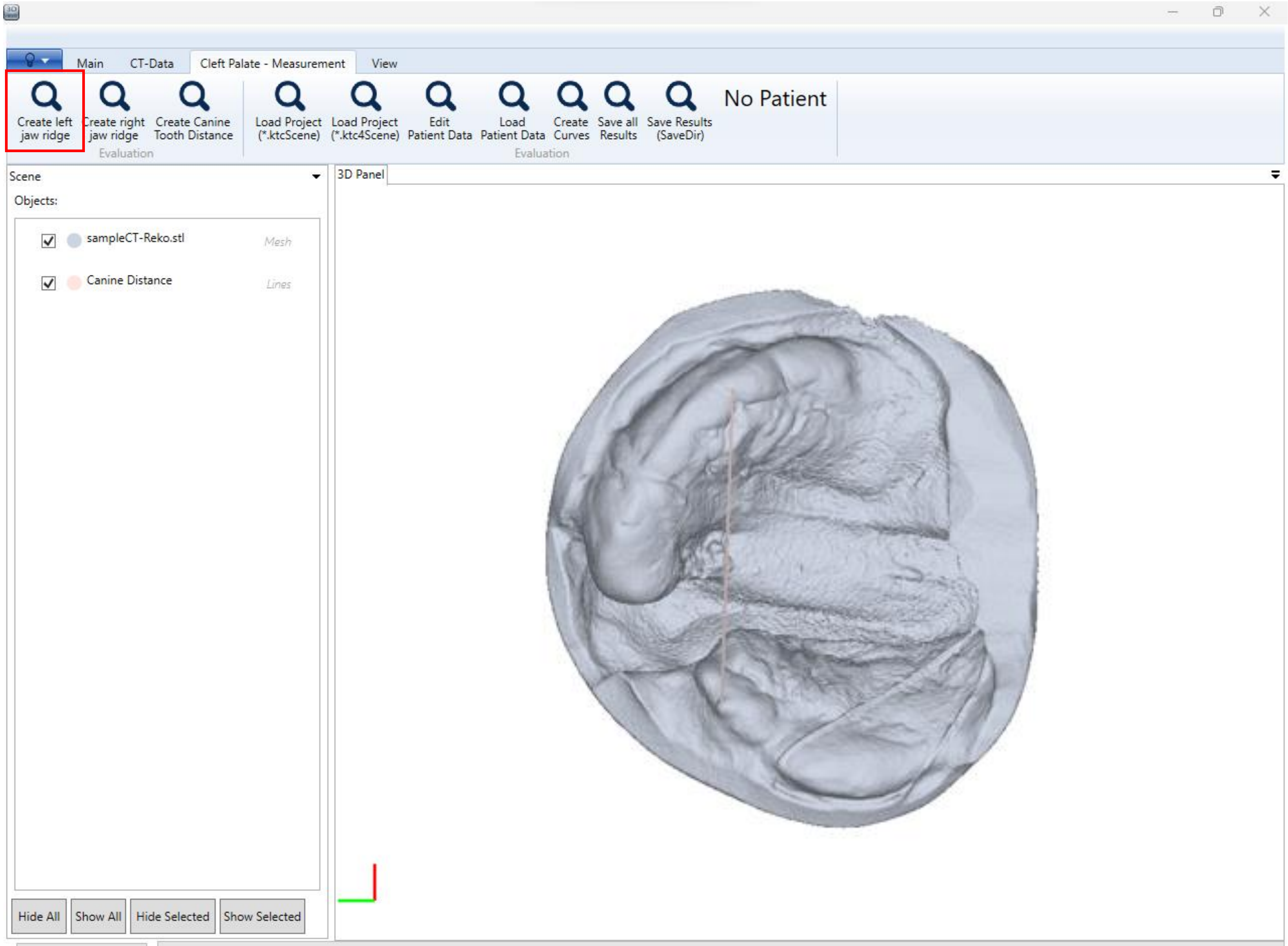
Main
CT-Data
Cleft Palate - Measurement
View
Create left jaw ridge
Create right jaw ridge
Create Canine Tooth Distance
Evaluation
Load Project (*.ktcScene)
Load Project (*.ktc4Scene)
Edit Patient Data
Load Patient Data
Create Curves
Save all Results
Save Results (SaveDir)
Evaluation
No Patient
Scene
Objects:
sampleCT-Reko.stl
Mesh
Canine Distance
Lines
3D Panel
Hide All
Show All
Hide Selected
Show Selected

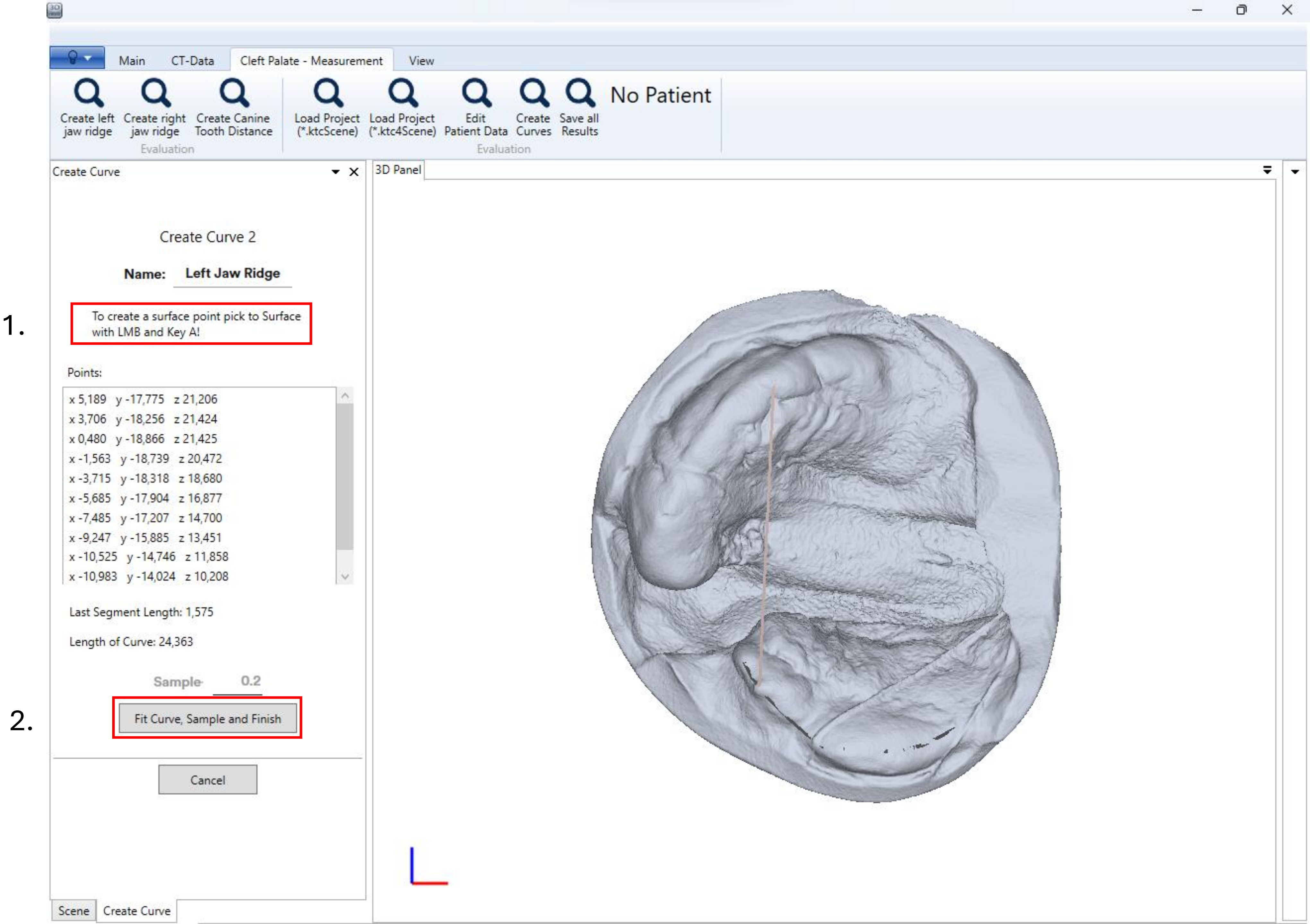
Main
CT-Data
Cleft Palate - Measurement
View
Create left jaw ridge
Create right jaw ridge
Create Canine Tooth Distance
Evaluation
Load Project (*.ktcScene)
Load Project (*.ktc4Scene)
Edit Patient Data
Create Curves
Save all Results
Evaluation
No Patient
Create Curve
3D Panel
Create Curve 2
Name:
Left Jaw Ridge
1.
To create a surface point pick to Surface with LMB and Key A!
Points:
x 5,189 y -17,775 z 21,206
x 3,706 y -18,256 z 21,424
x 0,480 y -18,866 z 21,425
x -1,563 y -18,739 z 20,472
x -3,715 y -18,318 z 18,680
x -5,685 y -17,904 z 16,877
x -7,485 y -17,207 z 14,700
x -9,247 y -15,885 z 13,451
x -10,525 y -14,746 z 11,858
x -10,983 y -14,024 z 10,208
Last Segment Length: 1,575
Length of Curve: 24,363
Sample
0.2
2.
Fit Curve, Sample and Finish
Cancel
Scene
Create Curve

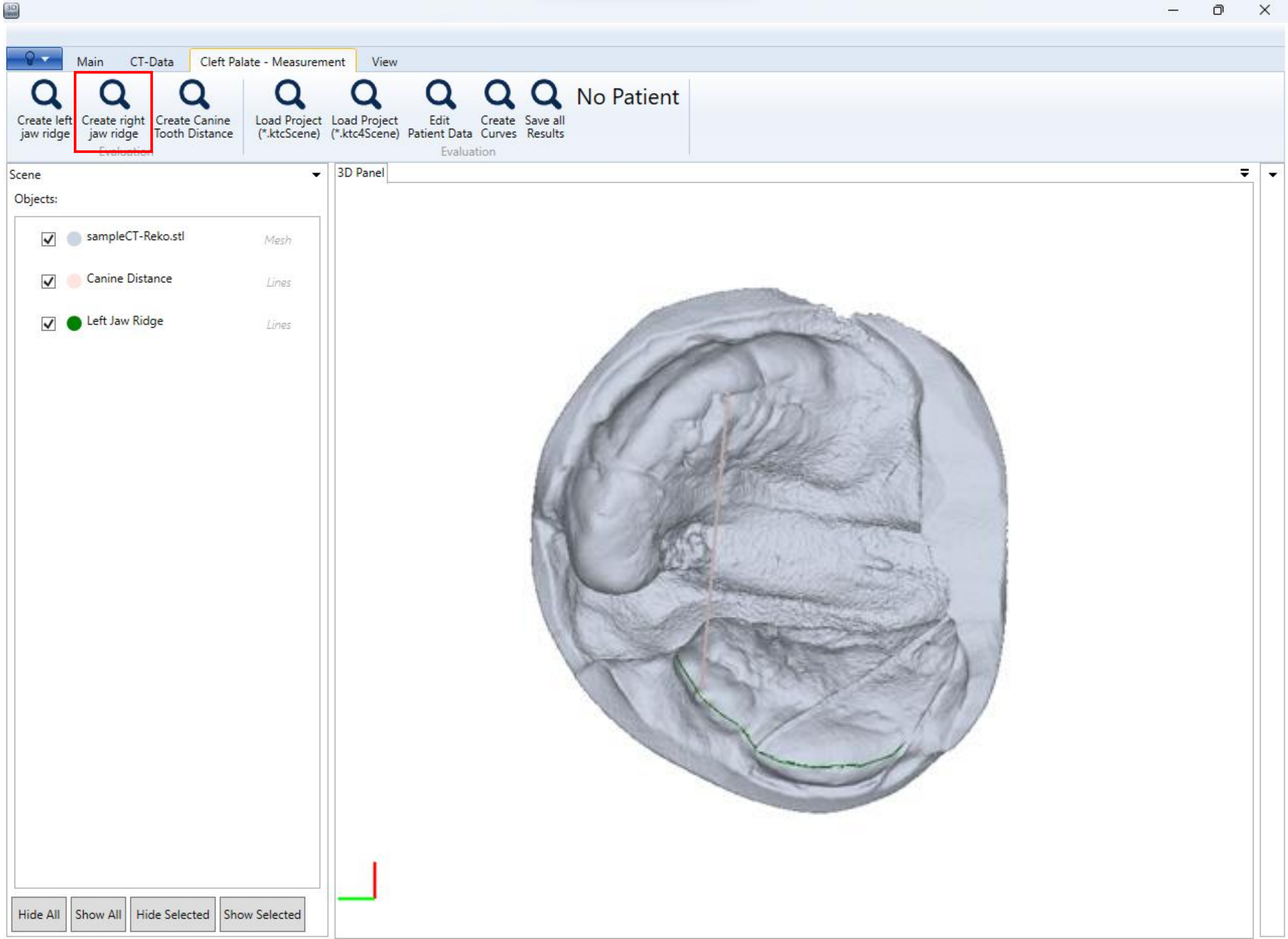
Main
CT-Data
Cleft Palate - Measurement
View
Create left jaw ridge
Create right jaw ridge
Create Canine Tooth Distance
Load Project (*.ktcScene)
Load Project (*.ktc4Scene)
Edit Patient Data
Create Curves
Save all Results
No Patient
Evaluation
Scene
Objects:
sampleCT-Reko.stl
Mesh
Canine Distance
Lines
Left Jaw Ridge
Lines
3D Panel
Hide All
Show All
Hide Selected
Show Selected

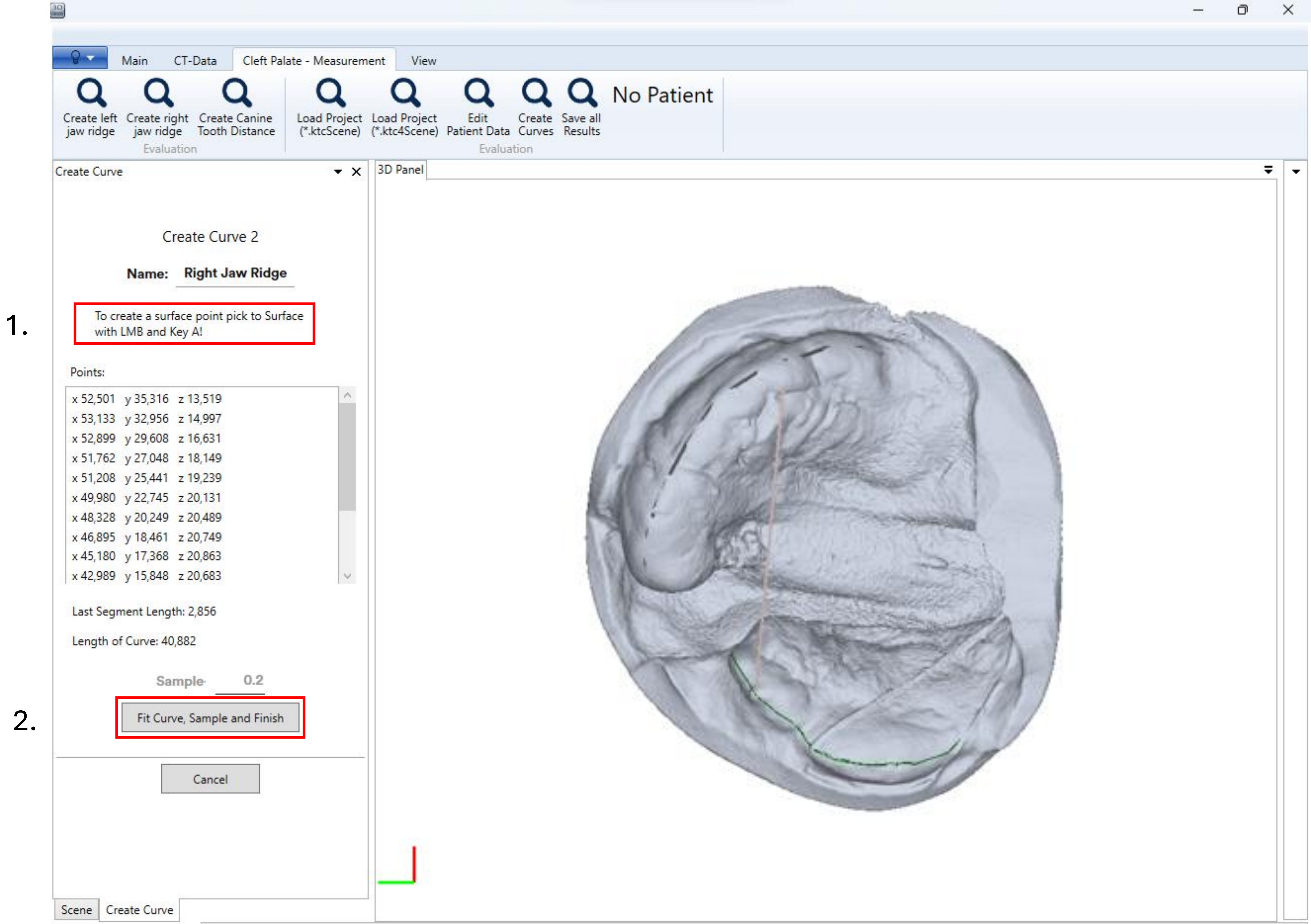
Main
CT-Data
Cleft Palate - Measurement
View
Create left jaw ridge
Create right jaw ridge
Create Canine Tooth Distance
Evaluation
Load Project (*.ktcScene)
Load Project (*.ktc4Scene)
Edit Patient Data
Create Curves
Save all Results
Evaluation
No Patient
Create Curve
3D Panel
Create Curve 2
Name: Right Jaw Ridge
1.
To create a surface point pick to Surface with LMB and Key A!
Points:
x 52,501 y 35,316 z 13,519
x 53,133 y 32,956 z 14,997
x 52,899 y 29,608 z 16,631
x 51,762 y 27,048 z 18,149
x 51,208 y 25,441 z 19,239
x 49,980 y 22,745 z 20,131
x 48,328 y 20,249 z 20,489
x 46,895 y 18,461 z 20,749
x 45,180 y 17,368 z 20,863
x 42,989 y 15,848 z 20,683
Last Segment Length: 2,856
Length of Curve: 40,882
Sample 0.2
2.
Fit Curve, Sample and Finish
Cancel
Scene
Create Curve

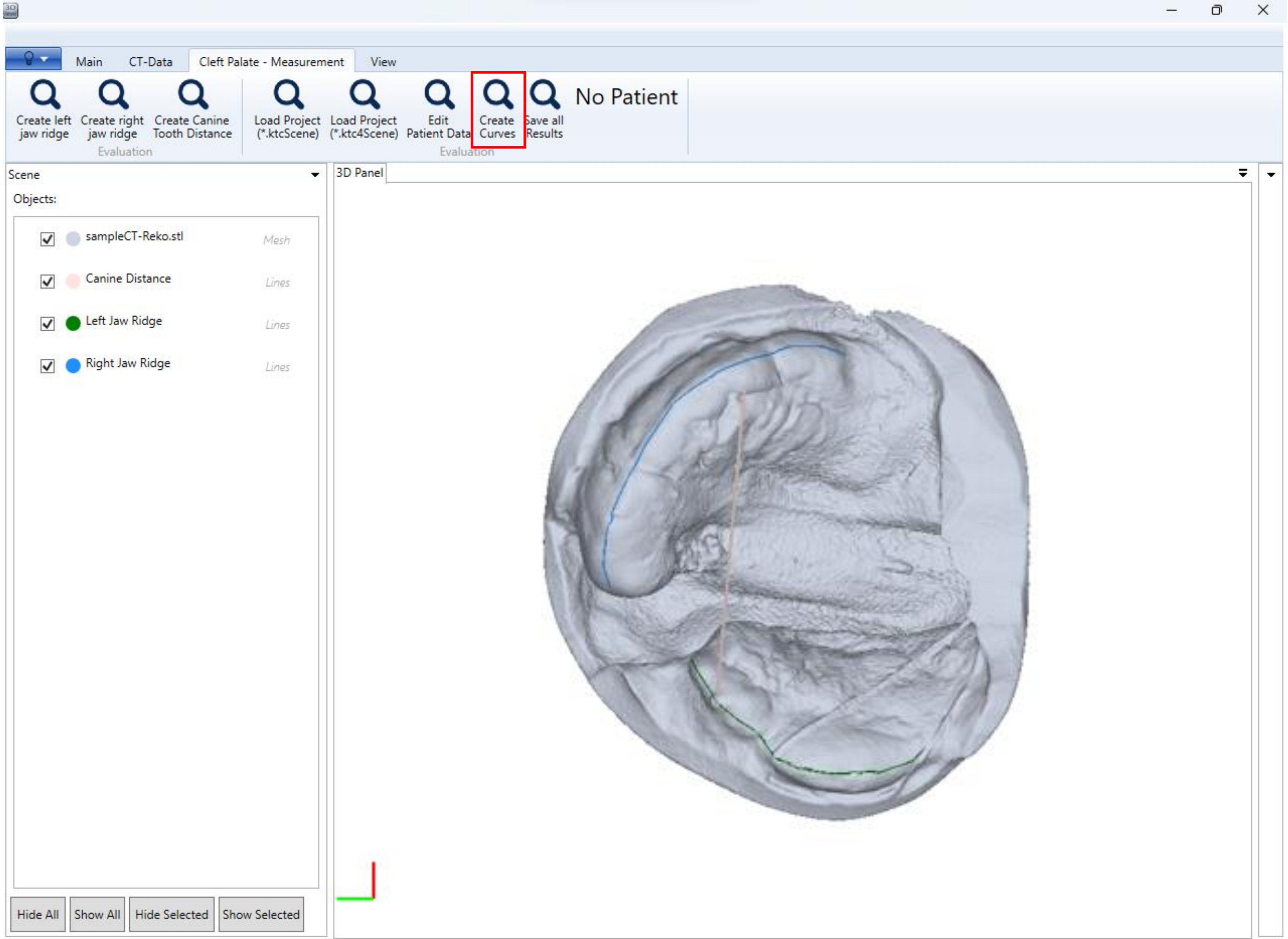

Main
CT-Data
Cleft Palate - Measurement
View
Create left jaw ridge
Create right jaw ridge
Create Canine Tooth Distance
Evaluation
Load Project (*.ktcScene)
Load Project (*.ktc4Scene)
Edit Patient Data
Create Curves
Save all Results
Evaluation
No Patient
Scene
Objects:
sampleCT-Reko.stl
Mesh
Canine Distance
Lines
Left Jaw Ridge
Lines
Right Jaw Ridge
Lines
Hide All
Show All
Hide Selected
Show Selected
3D Panel

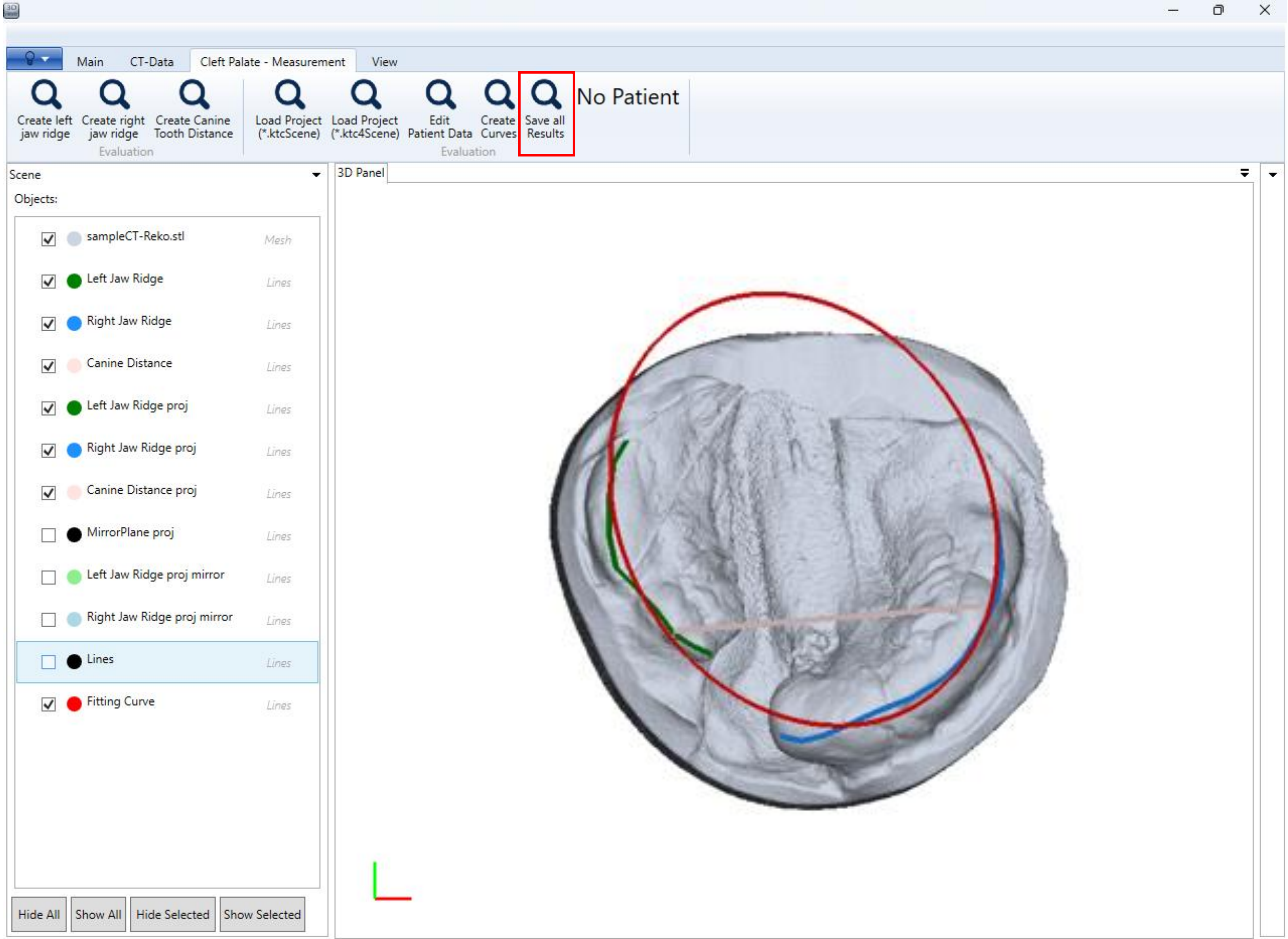
Main
CT-Data
Cleft Palate - Measurement
View
Create left jaw ridge
Create right jaw ridge
Create Canine Tooth Distance
Evaluation
Load Project (*.ktcScene)
Load Project (*.ktc4Scene)
Edit Patient Data
Create Curves
Save all Results
Evaluation
No Patient
Scene
Objects:
sampleCT-Reko.stl
Mesh
Left Jaw Ridge
Lines
Right Jaw Ridge
Lines
Canine Distance
Lines
Left Jaw Ridge proj
Lines
Right Jaw Ridge proj
Lines
Canine Distance proj
Lines
MirrorPlane proj
Lines
Left Jaw Ridge proj mirror
Lines
Right Jaw Ridge proj mirror
Lines
Lines
Lines
Fitting Curve
Lines
Hide All
Show All
Hide Selected
Show Selected
3D Panel

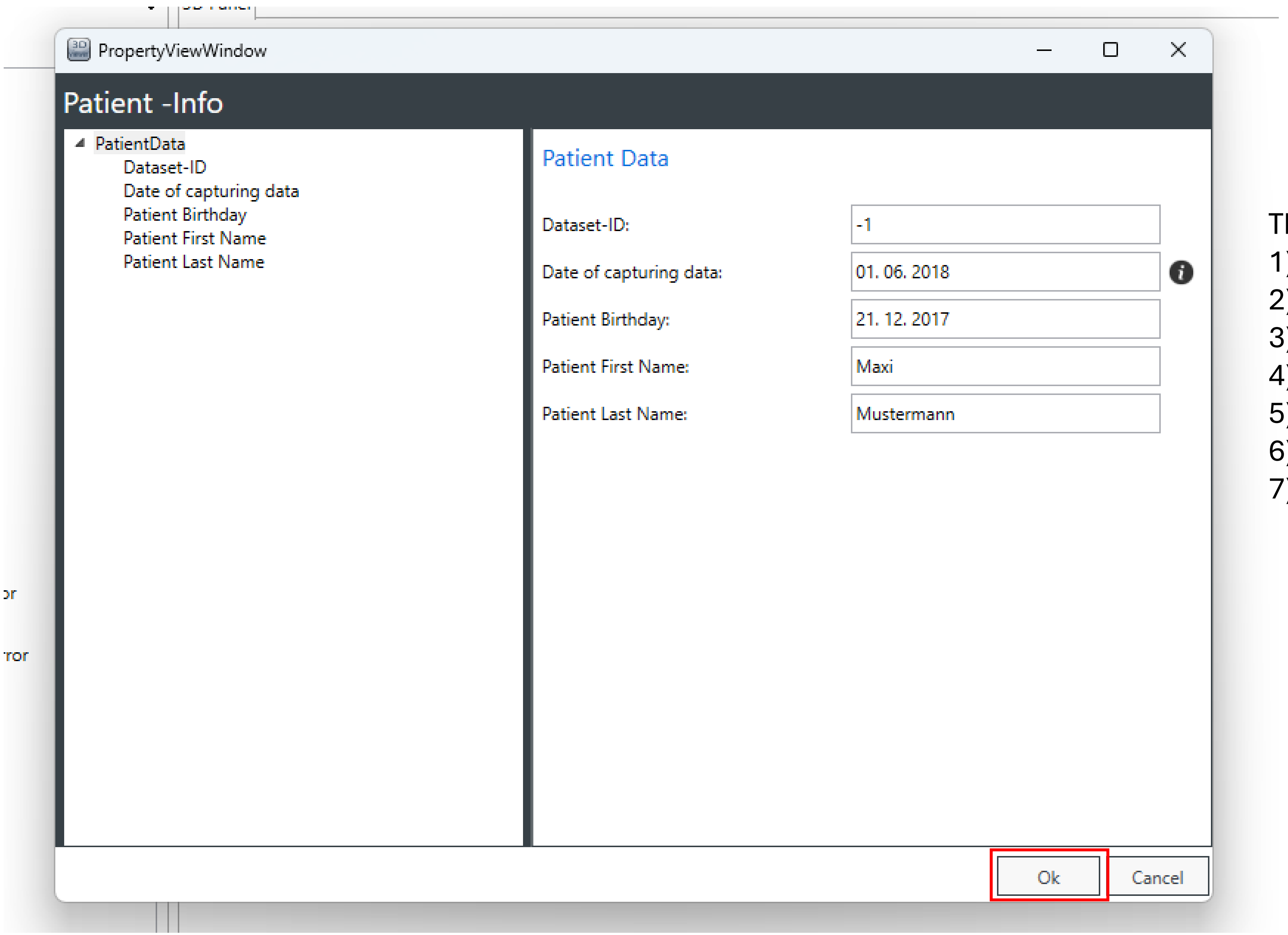

The following files are created and saved:

1) *_patient.xml
2) *.ktc4Scene
3) *_curves.csv
4) *_curves.xml
5) *_fitt.xml
6) *_overview.png
7) *_ALL.png

The *_overview.png file contains the measurement data for the alveolar ridge length.

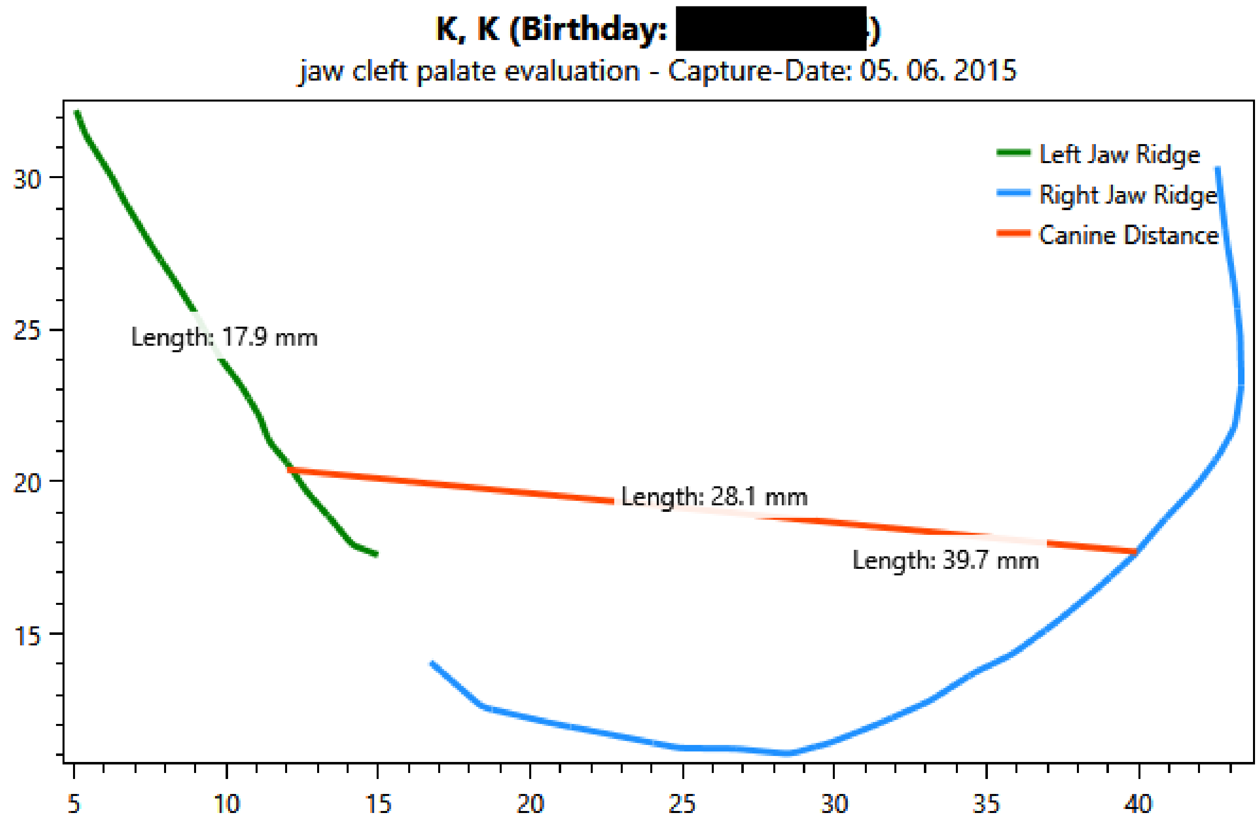

The *_fitt.xml file contains the measurement data for calculating the Q coefficient.


```
<?xml version="1.0"?>
<QuadraticCurveFittingResult
xmlns:xsd="http://www.w3.org/2001/XMLSchema"
xmlns:xsi="http://www.w3.org/2001/XMLSchema-instance">
  <G0>0.9982864097549840б</C0>
  <G1>-0.042661848864092689</C1>
  <G2>-0.040043538933258134</C2>
  <G3>0.00066502291747489589</C3>
  <G4>0.00048133871694460567</C4>
  <G5>0.00022759011462240158</C5>
  <QuadraticCurveTyp>Ellipse</QuadraticCurveTyp>
  <Quality>0.00013265174768868924</Quality>
  <UseResult1>false</UseResult1>
</QuadraticCurveFittingResult>
```
